# Tau interactome mapping based identification of Otub1 as Tau deubiquitinase involved in accumulation of pathological Tau forms in vitro and in vivo

**DOI:** 10.1007/s00401-016-1663-9

**Published:** 2017-01-12

**Authors:** Peng Wang, Gerard Joberty, Arjan Buist, Alexandre Vanoosthuyse, Ilie-Cosmin Stancu, Bruno Vasconcelos, Nathalie Pierrot, Maria Faelth-Savitski, Pascal Kienlen-Campard, Jean-Noël Octave, Marcus Bantscheff, Gerard Drewes, Diederik Moechars, Ilse Dewachter

**Affiliations:** 1 Alzheimer Dementia Group, Institute of Neuroscience, Catholic University of Louvain, 1200 Brussels, Belgium; 2 Department of Neuroscience, Janssen Research and Development, A Division of Janssen Pharmaceutica NV, 2340 Beerse, Belgium; 3 Cellzome GmbH, Molecular Discovery Research, GlaxoSmithKline, Meyerhofstrasse 1, Heidelberg, Germany; 4 BioMedical Research Institute, Hasselt University, Hasselt, Belgium

**Keywords:** Tau, Tau oligomerization, Tau pathology, Alzheimer’s disease, Interactome mapping, Otub1, Ubiquitination

## Abstract

Dysregulated proteostasis is a key feature of a variety of neurodegenerative disorders. In Alzheimer’s disease (AD), progression of symptoms closely correlates with spatiotemporal progression of Tau aggregation, with “early” oligomeric Tau forms rather than mature neurofibrillary tangles (NFTs) considered to be pathogenetic culprits. The ubiquitin–proteasome system (UPS) controls degradation of soluble normal and abnormally folded cytosolic proteins. The UPS is affected in AD and is identified by genomewide association study (GWAS) as a risk pathway for AD. The UPS is determined by balanced regulation of ubiquitination and deubiquitination. In this work, we performed isobaric tags for relative and absolute quantitation (iTRAQ)-based Tau interactome mapping to gain unbiased insight into Tau pathophysiology and to identify novel Tau-directed therapeutic targets. Focusing on Tau deubiquitination, we here identify Otub1 as a Tau-deubiquitinating enzyme. Otub1 directly affected Lys48-linked Tau deubiquitination, impairing Tau degradation, dependent on its catalytically active cysteine, but independent of its noncanonical pathway modulated by its N-terminal domain in primary neurons. Otub1 strongly increased AT8-positive Tau and oligomeric Tau forms and increased Tau-seeded Tau aggregation in primary neurons. Finally, we demonstrated that expression of Otub1 but not its catalytically inactive form induced pathological Tau forms after 2 months in Tau transgenic mice in vivo, including AT8-positive Tau and oligomeric Tau forms. Taken together, we here identified Otub1 as a Tau deubiquitinase in vitro and in vivo, involved in formation of pathological Tau forms, including small soluble oligomeric forms. Otub1 and particularly Otub1 inhibitors, currently under development for cancer therapies, may therefore yield interesting novel therapeutic avenues for Tauopathies and AD.

**Electronic supplementary material:**

The online version of this article (doi:10.1007/s00401-016-1663-9) contains supplementary material, which is available to authorized users.

## Introduction

Alzheimer’s disease (AD) is the most prevalent form of dementia, being characterized by progressive accumulation of amyloid plaques and neurofibrillary tangles, composed of aggregated Aβ and hyperphosphorylated Tau, respectively [9, 69]. Over the last decade, Tau has emerged as an important therapeutic target. This is underscored by (i) the close correlation of Tau pathology with the progression of symptoms and (ii) the existence of a family of Tauopathies which are neurodegenerative disorders characterized by Tau aggregation spatiotemporally correlating with brain dysfunction and associated symptoms, as well as, most importantly, (iii) the identification of mutations in Tau causally linked to Tauopathies, demonstrating a causal executive role of Tau in Tauopathies. Although Tau aggregation is closely linked to symptom progression, smaller oligomeric Tau forms rather than mature full-blown neurofibrillary tangles (NFTs) are generally considered to be pathogenetic culprits in the disease process [6, 11, 12, 15, 20, 33, 37, 39, 40, 59, 60, 67, 70]. Accumulation of misfolded and aggregated proteins is a key feature of a variety of neurodegenerative disorders, raising interest in understanding clearance and degradation mechanisms of these pathogenetic culprits [7, 22, 28, 35, 42, 43]. The UPS selectively degrades normal and abnormally folded soluble proteins, which are tagged by ubiquitin for elimination [22], while the autophagic–lysosomal system (ALS) mainly degrades large protein aggregates or inclusions and organelles. With smaller soluble Tau forms considered to be toxic culprits, the UPS represents an important target for therapeutic interventions and is the main focus of this work.

The UPS is reported to be downregulated in various neurodegenerative disorders, with increased proteasome activity shown to be beneficial in related disease models [15, 28, 42, 52]. The presence of ubiquitinated Tau in pathological lesions of AD provided the first lead implicating the UPS in AD and Tauopathies [49, 50]. In AD patients’ brain, paired helical filaments (PHFs) were found to be associated with impaired proteasome activity in a brain-region specific way [15, 28, 34, 35]. Furthermore, a ubiquitin mutant called UBB^+1^, with a 19-AA extension, has been identified in neurons from AD patients and suppresses UPS function [38, 45, 71]. UBB^+1^-induced proteasome dysfunction resulted in Tau pathology and related neuronal dysfunction in transgenic mice expressing UBB^+1^ [29]. Accumulating evidence indicates that the UPS is implicated in clearance of different Tau forms and its dysfunction is closely related to Tau aggregation and accumulation [1, 13–16, 25, 46, 57, 68]. Conversely, Tau accumulation was recently shown to impair proteasomal degradation, suggesting a vicious circle [52]. Degradation of target proteins through the UPS is regulated by ubiquitination, with differently linked (Lys6, Lys11, Lys48, and Lys63) polyubiquitinated Tau identified in AD brains and models [13, 49–51, 56]. Different Tau forms can be ubiquitinated by E3 ligases CHIP [57, 63], TRAF6 [1], and MARCH7 [19], with particularly CHIP/Hsp70-dependent polyubiquitination characterized in detail in vitro and in vivo. Ubiquitination is highly dynamic and reversible by the action of deubiquitinating enzymes known as deubiquitinases (DUBs), hence being determined by balanced regulation between ubiquitination and deubiquitination. While Tau ubiquitination has been studied intensively, its deubiquitination remains less well explored.

Deubiquitinases are attracting increasing interest as therapeutic targets. Otub1 is an ovarian-tumor domain cysteine protease deubiquitinating enzyme [2] with strong preference for Lys48-linked polyubiquitin chains over Lys11-, Lys29-, or Lys63-linked polyubiquitin chains in the canonical pathway, dependent on its highly conserved active-site cysteine (C91) [17, 48, 74]. Recently, a noncanonical pathway of Otub1, involved in DNA double-strand break response by modulating Lys63 polyubiquitin chain formation, was highlighted [31, 53, 77, 78]. Otub1 has been implicated in a broad range of cellular pathways such as IL-1β-induced inflammation and stabilization of proteins including c-IAP1, p53, TRAF3/6, SMAD2/3, and RhoA, indicating that it is an important regulator in different physiological processes [23, 26, 32, 44, 66], while its role in the nervous system remains unknown.

In this study, we performed iTRAQ-based Tau interactome mapping to identify Tau-interacting proteins with Tau-modifying potential as therapeutic targets. This highlighted Otub1 as a potential entry point to better understand the link between the UPS and Tauopathies. We demonstrate here for the first time that Tau is deubiquitinated by Otub1. We demonstrate that Otub1 regulates levels of Lys48-linked ubiquitin-conjugated Tau and increases pathological forms of Tau in vitro and in vivo.

## Materials and methods

### Animals

TauP301S (PS19) mice [81] were bred and used in our laboratory as reported previously [64, 65, 72]. The mice bred in our laboratory develop Tau pathology and a neurodegenerative phenotype starting at around ~10–11 months. In this study, stereotactic injections were performed at P0 and age-matched littermates were used for analysis 2 months postinjection. At the age of 2 months, no Tau pathology or AT8-positive staining is detected in TauP301S mice in our colony. All experiments were performed in compliance with protocols approved by the UCLouvain Ethical Committee for Animal Welfare.

### Reagents and antibodies

MG132 and cycloheximide were purchased from Sigma. Phosphatase inhibitor (PhosSTOP™) and protease inhibitor cocktails (cOmplete™, Mini, EDTA-free) were obtained from Roche. Primary antibodies used in this study included antibodies directed against human Tau (Dako), Otub1, Ubiquitin-Lys48-specific, Ubiquitin-Lys63-specific (Abcam), Phospho-Tau (Ser202/Thr205) AT8, Phospho-Tau (Thr231) AT180, Phospho-Tau (Thr212/Ser214) AT100 (Thermo Fisher Scientific), and Oligomeric-Tau T22 (Merck), applied in combination with appropriate horseradish peroxidase (HRP) or Alexa Fluor® (488/568/647) coupled secondary antibodies.

### Cell culture and transfection

Human kidney-derived QBI-293 were originally obtained from QBiogene (Carlsbad, CA, USA) and HEK293 cells from American Type Culture Collection (ATCC), and were cultured according to the manufacturer’s protocol. All cells were maintained at 37 °C in humidified atmosphere with 5% CO_2_. Cells were transfected with plasmids expressing Otub1, USP9x, USP5 or empty vector using FuGENE® 6 (Roche) or short interfering RNAs (siRNAs) using Lipofectamine 2000 (Invitrogen) according to the manufacturer’s protocol. For analysis of the effects on Tau or on Tau-seeded Tau aggregation, transfection was performed 24 h before starting the assay.

### In vivo gene delivery

Adeno-associated viral (AAV) vectors expressing wild-type Otub1 fused with a green fluorescent protein (GFP) tag (AAV–Otub1) or expressing GFP (AAV–GFP) driven by neuron-specific Syn promoter were generated using a previously described plasmid (Addgene, plasmid #26976). Mutant Otub1 C91A and N-terminal truncation (N-T) were generated by molecular cloning starting from the WT-Otub1 construct. AAV viruses were produced in HEK-AAV cells using AAV-DJ8 kit (Cell Biolabs, Inc.), and subsequently purified and concentrated by iodixanol-based ultracentrifugation. AAV titer was tested using a QuickTiter™ AAV Quantitation Kit (Cell Biolabs, Inc). For P0 injection, each mouse was injected into the lateral ventricles of both cerebral hemispheres with 4.2 × 10^9^ total viral particles per side, TauP301S transgenic mice were euthanized, and brains were dissected as described previously following transcardial flushing and analyzed at 2 months postinjection [64, 65, 72].

### Immunoprecipitation and Western blot analysis

To detect Tau ubiquitination, primary neurons were infected with AAV–Otub1 (wild type; catalytically dead mutant C91A; N-terminal truncation) or AAV–GFP, five days after infection, cells were washed with phosphate-buffered saline (PBS) and lysed in TGN lysis buffer (50 mM Tris HCl, pH 7.5, 200 mM NaCl, 50 mM sodium β-glycerophosphate, 1% Tween 20, 0.2% NP40) containing phosphatase inhibitor (PhosSTOP™; Roche) and protease inhibitor cocktails (cOmplete™, Mini, EDTA-free; Roche) at 4 °C for 30 min on a wheel rotor, and spun at 12,000×*g* for 15 min. Following preclearing without antibodies at 4 °C for 1 h, the supernatants were incubated with specific antibodies for 1 h at 4 °C, followed by incubation with protein A-Sepharose beads at 4 °C for 45 min. Following stringent washing with TGN buffer and PBS, immunoprecipitated proteins were analyzed by immunoblotting.

For Western blot analysis, cells were washed twice with PBS and extracted for 30 min at 4 °C with Triton lysis buffer (1% Triton X-100, 50 mM Tris, 150 mM NaCl, pH 7.6) containing protease and phosphatase inhibitors, and centrifuged at 12,000×*g* for 15 min at 4 °C to remove insoluble material. Protein content was determined by BCA Protein Assay kit (Thermo Fisher Scientific, Waltham, MA, USA). Samples (10 μg) were separated using precast 8% Tris–glycine gels or 4–12% Bis–Tris gels (MOPS running; Invitrogen) and transferred to polyvinylidene difluoride membranes. Immunoblotting was performed using the indicated primary antibodies with corresponding secondary antibodies, and developed using ECL kit (PerkinElmer, Waltham, MA, USA).

### Tau aggregation assay

Tau PFFs (synthetic preformed fibrils), referred to as “Tau seeds,” were generated as described previously [24, 65, 72]. Truncated human Tau fragments bearing a proaggregation mutant (Tau-P301L) containing the four-repeat domain [K18; Q244-E372 (4RTau)], N-terminally Myc-tagged were produced in *Escherichia coli* (TEBU-Bio). Tau-PFFs were obtained by incubation of Tau fragments (66 μM) at 37 °C for 5 days in presence of heparin (133 μM) in 100 mM ammonium acetate buffer (pH 7.0), spun down (100,000×*g*, 1 h, 4 °C), resuspended to 333 μM, and sonicated before use.

In vitro Tau aggregation assay in HEK293 cells: PFF-induced Tau aggregation in vitro was performed essentially as described previously [24, 65, 72]. Sonicated Tau seeds were diluted in 100 mM ammonium acetate buffer (pH 7.0) to 10 µM solution and sonicated with 8 pulses/30% amplitude and added to the cells using BioPORTER® (AMS Biotechnology, Milton, UK) according to previously described protocol. To detect Tau aggregation, cells were fixed with 4% paraformaldehyde (PFA) containing 2% sucrose and 1% Triton X-100 for 15 min to remove soluble proteins. After washing with PBS, aggregated Tau-GFP was analyzed microscopically. To detect the effect of Otub1, USP5, and USP9x overexpression or knockdown on Tau aggregation, stably Tau-expressing QBI-293 cells were transfected with plasmid and empty vector or siRNA in 24-well plates. At 24 h after transfection, the growth medium was replaced with OptiMEM, and sonicated Tau seeds (10 μM) were added to BioPORTER® single-use tubes and added to cells. Three days after seeding, Tau-GFP aggregation was measured by microscopy.

In vitro Tau aggregation assay in primary neurons was performed as described previously [65]. Tau seeds (10 nM) were added at DIV3 and DIV6 to primary cortical neuronal cultures (PNC) from P0 heterozygous TauP301S pups. To detect the effect of Otub1 on Tau aggregation in primary neurons, AAV–Otub1 and AAV–GFP infections were carried out at DIV3, Tau seeds (10 nM) were added at DIV6 and DIV9, and cells were fixed at DIV12 with 4% PFA containing 2% sucrose and 1% Triton X-100 for 15 min to remove soluble proteins. After washing with PBS, aggregated Tau-GFP was detected under microscope.

### Immunofluorescence microscopy assay

At indicated times after infection, cells were washed by phosphate-buffered saline (PBS) for three times and fixed with 4% paraformaldehyde in PBS for 15 min at room temperature (RT). For stringent extraction to detect aggregated Tau, 1% Triton X-100 (4% PFA, 2% sucrose) was used. Cells were briefly permeabilized in PBS/0.2% Triton X-100, then blocked in blocking solution (PBS containing 10% fetal calf serum and 0.1% Triton X-100). Primary and secondary antibody incubations were performed in blocking solution overnight at 4 °C or 1 h at RT using the indicated antibodies and goat anti-mouse IgG1 or goat anti-rabbit IgG1 secondary antibody coupled to Alexa^®^ 488, Alexa^®^ 568 or Alexa^®^ 647. Cells were visualized using a digital inverted fluorescence microscope (EVOS-xl auto microscope).

### Tau interactome mapping

Total mouse brain homogenates were used for coimmunoprecipitation in combination with three different polyclonal anti-Tau antibodies and control antibodies that were cross-linked to Sepharose beads. Mass spectrometry procedures were performed essentially as previously reported [4]. A detailed description of the Tau interactome mapping procedure is provided in the Supplemental Experimental Procedures.

## Results

### Tau interactome mapping identifies Tau-interacting proteins in mouse brain, including Otub1 as a potential Tau modifier

**Fig. 1:**
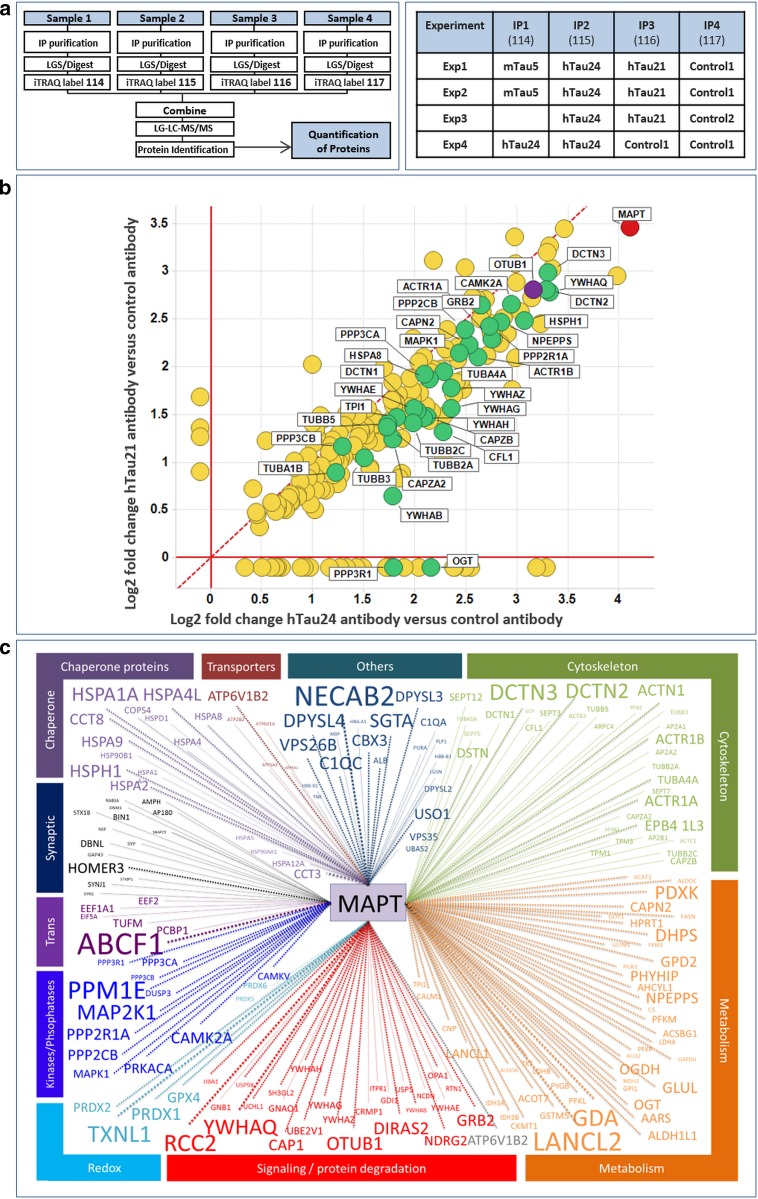
iTRAQ mass spectrometry-based Tau interactome mapping in mouse brain identifies well-characterized and novel Tau-interacting proteins. **a** Four immunoprecipitation (IP) experiments were performed using three anti-Tau and two control antibodies as indicated (*left panel* iTRAQ experimental layout; *right panel* detail of the four experiments performed). **b** Graphical display of anti-Tau immunoprecipitation (IP) data. Results of anti-Tau hTau24 (*X*-axis) IPs are compared with results of anti-Tau hTau21 IPs (*Y*-axis). Results are shown as log_2_ of ratio between quantification of protein immunoprecipitated with anti-Tau and control antibodies. Only proteins specifically precipitated with anti-Tau antibodies are displayed [known Tau interactors (*green dots*); Otub1 (*purple dot*)]. Significantly enriched proteins with only one of the two antibodies are artificially drawn below the axes. Statistical tools were applied to identify a list of Tau-interacting proteins from the differential co-IP analysis in the four experiments. **c** Following gene ontology analysis, candidate Tau interactors were grouped according to their function; a weighted (font size) presentation of the interactors (gene nomenclature) is displayed. The complete list of Tau-interacting proteins is provided in Tables S1–S4, and STRING analysis in Fig. S1

To identify Tau-interacting proteins with Tau-modifying potential, we performed Tau interactome mapping of endogenous Tau in mouse brain (Fig. 1) using a proteomic approach [3, 5]. Hereto, iTRAQ-based quantitative mass spectrometry was used in combination with differential coimmunoprecipitation of endogenous Tau from mouse brain. The use of isobaric isotope labels allows side-by-side quantitative comparison of up to four different samples [76] (Fig. 1a). Four Tau immunoprecipitation experiments were performed using three different anti-Tau antibodies and two different control non-Tau-capturing antibodies for differential analysis (Fig. 1a). Statistical tools were applied to score proteins for selective coprecipitation with Tau. Retrieval of several well-characterized Tau-interacting proteins validated the experimental approach (Fig. 1b). These included several tubulin isoforms [41, 75, 79], different components of the dynactin complex [47], enzymes modifying Tau phosphorylation, and members of the heat shock protein family, i.e., Hsc70 (Hspa8), Hsp70 (Hspa4), and Hsp110 (Hsp110) [18, 30, 57]. In addition, we identified many new putative Tau interactors, based on their significant enrichment in anti-Tau immunoprecipitation compared with control immunoprecipitations (Tables S1, S2). The full Tau interactome is presented in Supplementary Tables S1–S4. Gene ontology and STRING analysis were performed (Fig. S1; Table S3), and candidate interactors grouped according to their function (Fig. 1c; Table S4) to yield new insights into the pathophysiological role of Tau. Within this project, we focused on proteins involved in regulation of Tau ubiquitination and its degradation by the ubiquitin proteasome system, generally considered to be involved in degradation of small soluble proteins. We focused particularly on Tau deubiquitination and identified three major candidate deubiquitinating enzymes, including Otub1, USP5, and USP9x, with Otub1 as the strongest Tau interactor according to statistical analysis.

### Otub1 increases Tau stability and increases Tau aggregation in a well-characterized cellular Tau aggregation model

**Fig. 2:**
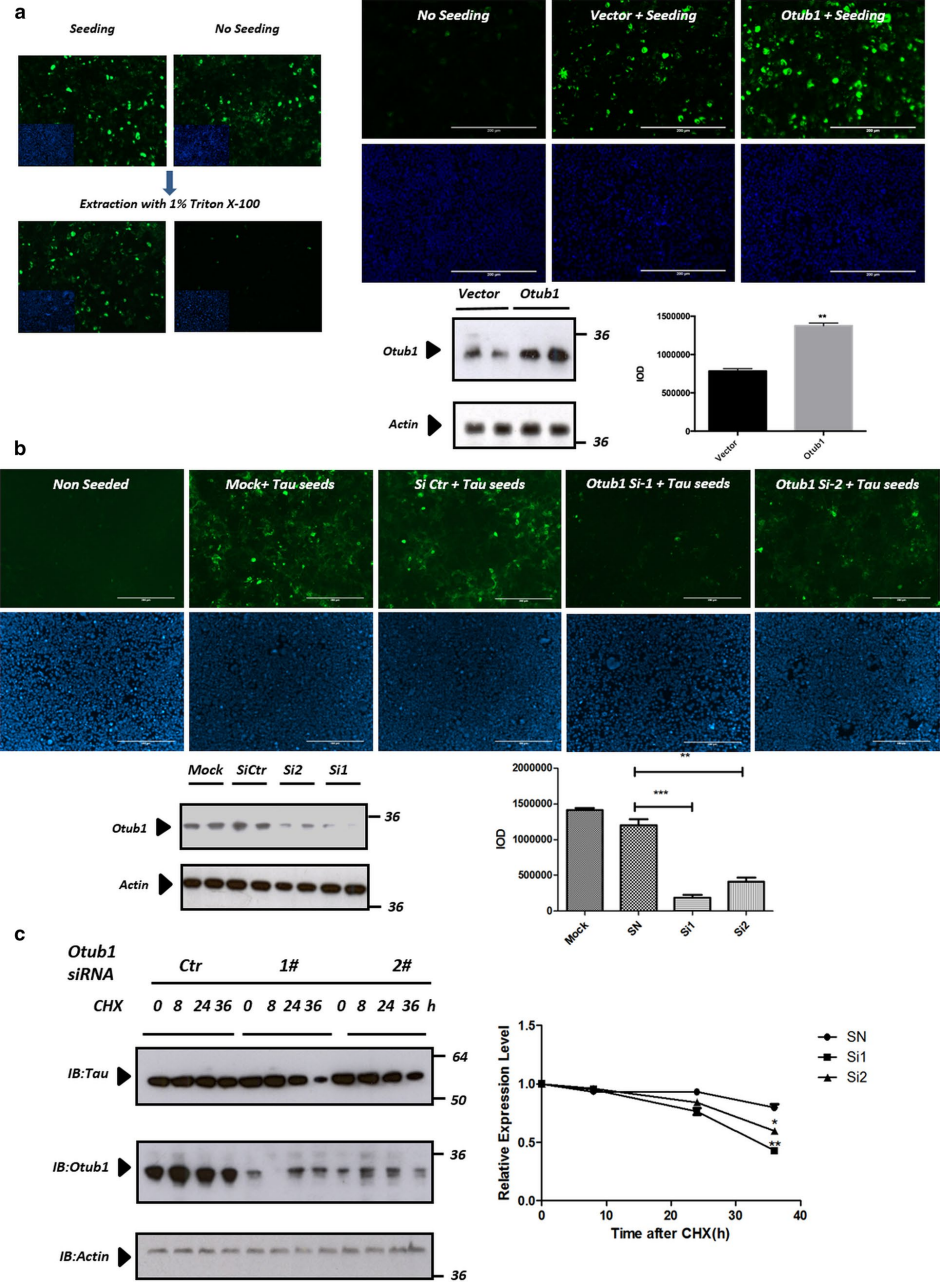
Otub1 increases Tau stability and increases Tau aggregation in a well-characterized cellular Tau aggregation model. **a** QBI-293 cells, stably expressing GFP-tagged TauP301L, were seeded with preaggregated synthetic Tau seeds to induce monomeric Tau aggregation. Tau aggregation was assessed by stringent extraction using 1% Triton X-100 to remove soluble proteins. Tau aggregation following expression of Otub1 and control vector in QBI-293 cells reveals significant increase of Tau aggregation following Otub1 expression, demonstrated by quantitative analysis (*n* > 36 fields per condition; *scale bar* 200 μm; **p value <0.01). Otub1 overexpression was biochemically validated using anti-Otub1 antibody. **b** Endogenous Otub1 depletion using two different siRNAs of Otub1 significantly inhibited Tau aggregation, correlating with the level of knockdown efficiency, as demonstrated by quantitative analysis (*n* > 36 fields per condition; *scale bar* 200 μm; **p value <0.01; ***p value <0.001). Otub1 knockdown efficiency was quantified by Western blotting using anti-Otub1 antibody. **c** Endogenous Otub1 depletion using two different siRNAs of Otub1 significantly increased Tau turnover, measured following treatment with cycloheximide (CHX, 20 μg/ml) for indicated times to inhibit protein synthesis and analyzed by immunoblotting. Actin served as loading control within each group (*p value <0.05; **p value <0.01)

To analyze whether Tau-interacting proteins, identified by the Tau interactome mapping, exerted modifying effects on Tau aggregation, we used a well-validated cellular Tau seeding model [24, 65, 72]. We analyzed the effect of the three identified candidate DUBs on Tau aggregation in vitro. In this assay, QBI cells stably expressing the longest human Tau isoform (2N4R), with the aggregation-prone P301L mutation and GFP tagged, were used in combination with preformed preaggregated synthetic Tau seeds consisting of Tau fragments harboring P301L mutant (myc-K18/P301L) to induce monomeric Tau aggregation. In this assay, addition of Tau seeds induces robust aggregation of monomeric human Tau, as assessed by cytological analysis following permeabilization with 1% Triton X-100 to remove soluble forms of Tau while retaining aggregated hyperphosphorylated Tau (Fig. 2a), as previously demonstrated and confirmed biochemically (Fig. S2) [65, 72]. Expression of the candidate deubiquitinases Otub1, USP5, and USP9x in this assay demonstrated significantly increased Tau aggregation following expression of Otub1 (Fig. 2a; Fig. S3), not observed following expression of USP5 or USP9x (Fig. S3). Next, we knocked down Otub1 in the Tau seeding assay, using two siRNAs of Otub1 with different knockdown efficiency. Endogenous Otub1 depletion significantly inhibited Tau aggregation, with the level of inhibition correlating with the level of knockdown efficiency, while use of a control siRNA did not affect levels of Otub1 nor aggregation efficiency (Fig. 2b). Combined, these data indicate that Otub1 affects Tau aggregation in a well-characterized Tau-seeded Tau aggregation model.

Since Otub1 is a deubiquitinase, known to regulate the stability of many crucial proteins in different cellular processes [23, 26, 32, 44, 66], we assessed whether Otub1 affected Tau stability by measuring Tau half-life following addition of the protein synthesis inhibitor cycloheximide (CHX). Knockdown of Otub1 increased Tau turnover, correlating with the decrease in expression of Otub1 obtained by the different siRNAs (Fig. 2c). Taken together, our data indicate that Otub1, a deubiquitinating enzyme, is a novel positive regulator of Tau aggregation and Tau stability in vitro, in a nonneuronal cell line.

### Otub1 increases total Tau concentration and Tau phosphorylation in primary neurons

**Fig. 3:**
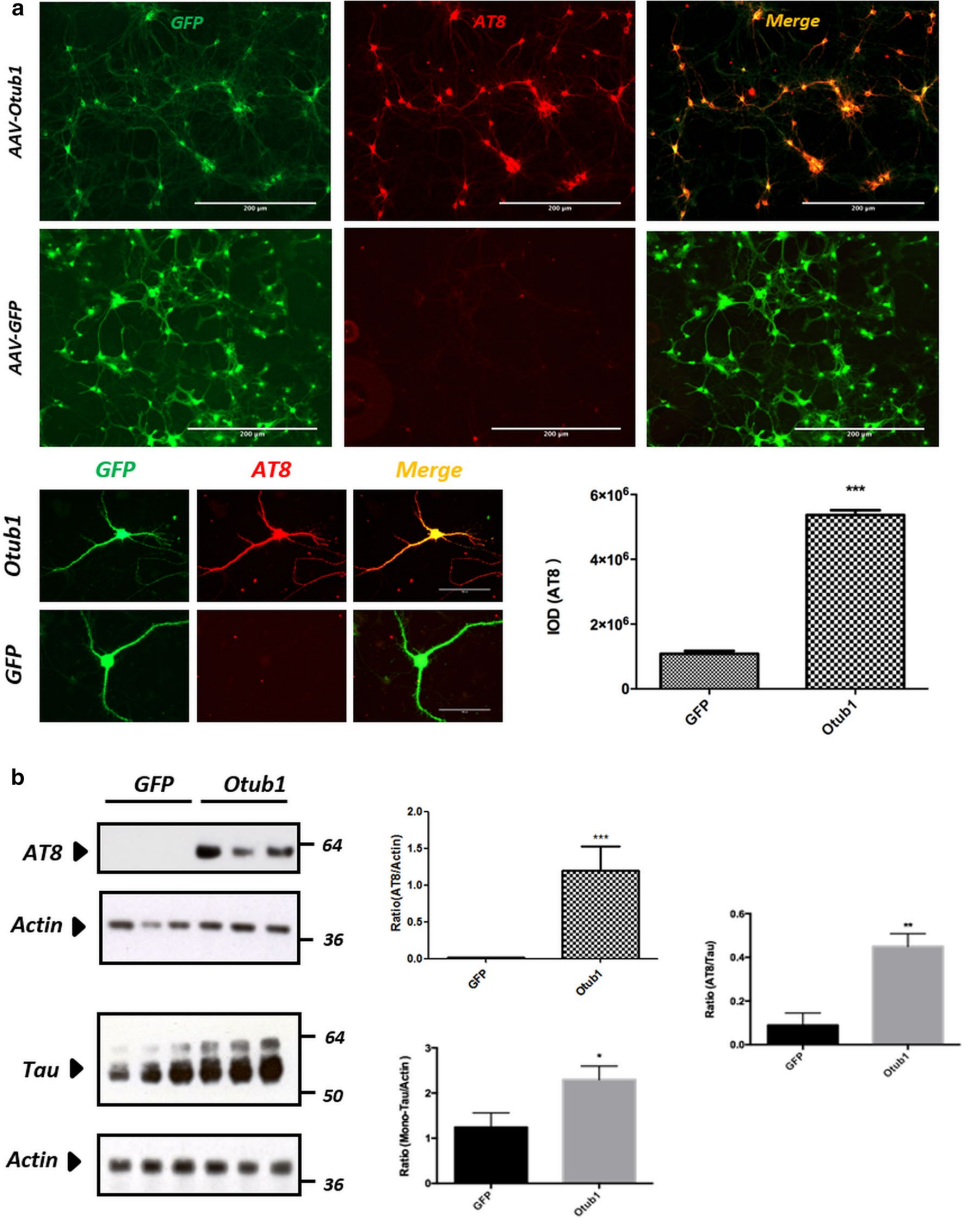
Otub1 increases total Tau concentration and Tau phosphorylation in primary neurons. **a** Immunofluorescence staining of primary TauP301S neurons infected with AAV–Otub1 or AAV–GFP using AT8 recognizing Tau phosphorylated at the pathologically relevant epitope Ser202/Thr205. Higher magnifications of AT8-stained neurons of Otub1- and GFP-expressing neurons are presented. Quantification of staining intensities are presented as mean ± standard error of the mean (SEM) (***p value <0.001; *n* = 3 different infections; for each infection, three repetitions are analyzed). **b** Western blot analysis of AAV-infected primary TauP301S neuron lysates using AT8 and total Tau antibodies. Actin was used as loading control on each blot. Ratio of AT8/Tau indicates the preferential effect of Otub1 on Phospho-Tau (AT8) (*p value <0.05; **p value <0.01; ***p value <0.001; *n* = 3 different infections; for each infection, three repetitions were analyzed)

We next analyzed the modulatory effect of Otub1 on Tau in primary neurons, as relevant cell type for the study of Tauopathies and AD. Hereto, primary cortical neurons, derived from TauP301S transgenic pups (PS19) [65, 81], were infected with AAV–Otub1–GFP or AAV–GFP driving neuron-specific expression. Immunocytological analysis with AT8 antibody against phosphorylated Tau (Ser202/Thr205) demonstrated that Otub1 expression drastically increased phospho-Tau (p-Tau Ser202/Thr205) in primary neurons compared with GFP expression (Fig. 3a). Biochemical analysis confirmed a sharp increase of phosphorylated Tau at Ser202/Thr205 in Otub1-infected primary neurons (Fig. 3b). Otub1 also significantly increased total Tau protein level compared with GFP control (Fig. 3b). This increase was, however, less robust than the increase in AT8-positive Tau, as reflected by the ratio of AT8/total Tau. Taken together, we conclude that overexpressed Otub1 in primary neurons expressing TauP301S increased total Tau and induced a robust increase in AT8-positive Tau.

### Otub1 enhances Tau oligomerization and Tau-seeded Tau aggregation in primary neurons

**Fig. 4:**
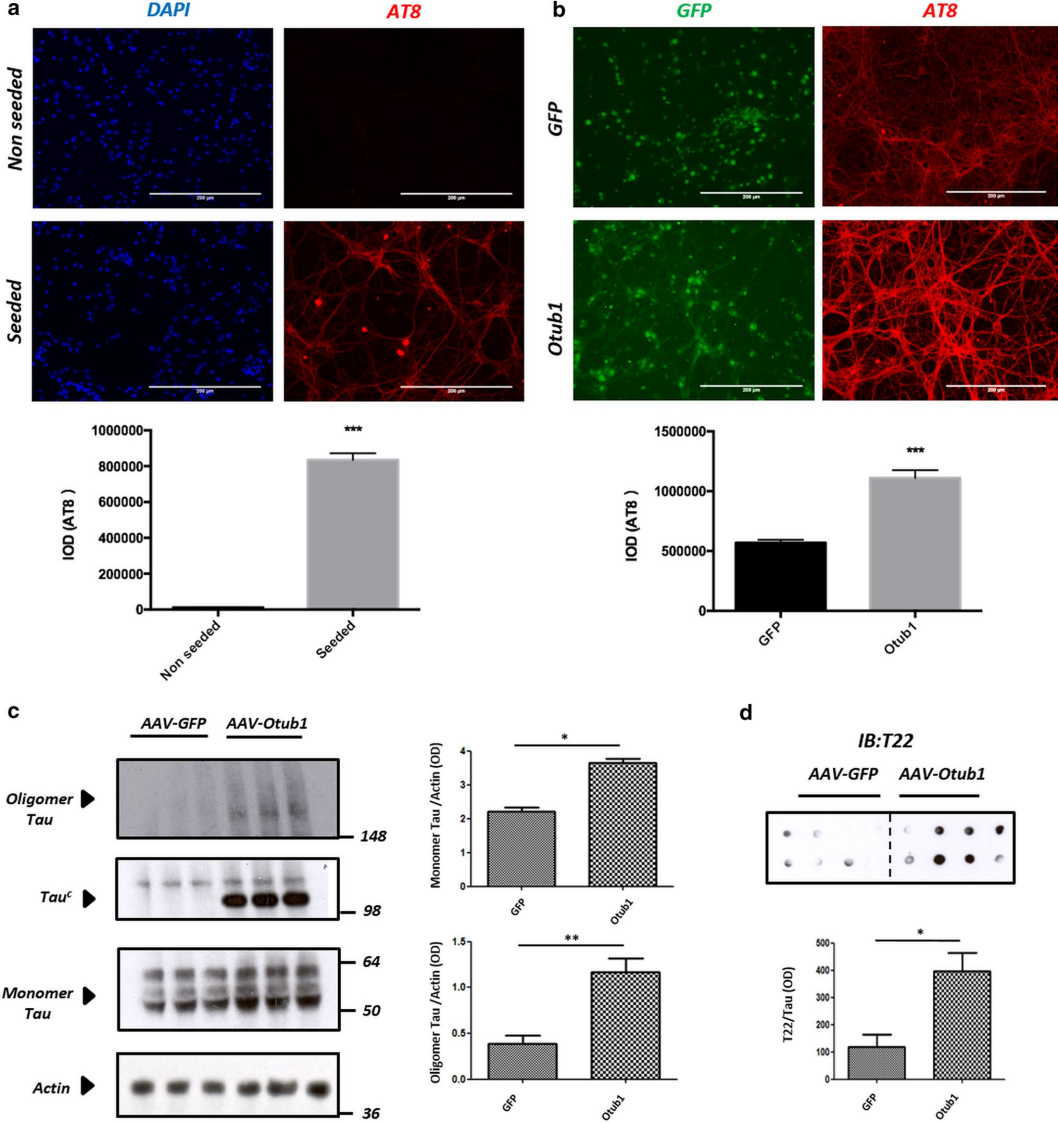
Otub1 enhances Tau oligomerization and Tau-seeded Tau aggregation in primary neurons. **a** Tau aggregation assay in TauP301S primary neurons. Addition of Tau seeds resulted in phosphorylated Tau (Ser202/Thr205) staining in soma and neurites, resisting stringent extraction with 1% Triton X-100 (*n* > 36 fields per condition; *scale bar* 200 μm; ***p value <0.001). **b** Primary TauP301S neurons were infected with AAV–Otub1 or AAV–GFP three days after culturing, and seeded 1 and 4 days postinfection, respectively. At 7 days after transduction, neurons were fixed and stringently extracted for analysis of aggregated Tau. Immunofluorescent staining using AT8 (Ser202/Thr205) after extraction is shown, demonstrating Tau aggregation. Quantification is presented as mean ± SEM (*n* = 3 different experiments; for each infection, 36 fields per condition were analyzed; *scale bar* 200 μm; ***p value <0.001). **c** Western blotting analysis of primary neuron extracts in nonreducing conditions, following AAV-driven expression of Otub1 and GFP, reveals increased monomeric Tau and increased oligomeric forms of Tau (detected as high-MW smears) in Otub1-overexpressing neurons (*n* = 3 different experiments; for each infection, three repetitions were analyzed; *p value <0.05; **p value <0.01). Induction of a Tau complex (homo- or heteromeric)—denoted Tau^c^—is clearly noticed following AAV–Otub1 but not AAV–GFP infection. **d** Dot-blot analysis of primary neuron lysates using oligomeric Tau-specific antibody T22 was performed and quantitatively analyzed following normalization to total Tau (dot-blot analysis) (mean ± SEM; *n* = 8 infection for each group; *p value <0.05)

We next analyzed the effect of Otub1 in a validated Tau aggregation assay in primary neurons [65, 81]. Addition of Tau seeds allows bypass of the lag phase of Tau aggregation, resulting in recruitment of soluble Tau into insoluble Tau aggregates, reflected in a robust detergent-resistant AT8 (phosphorylated Tau) signal throughout soma and neurites (Fig. 4a, Fig. S2a). We used this well-characterized assay to analyze a potential effect of Otub1 on Tau aggregation in TauP301S cortical primary neurons using AAV-driven expression of Otub1 or GFP for control. Otub1 expression strikingly enhanced detergent-resistant AT8-positive Tau accumulation compared with GFP-infected neurons (Fig. 4b), indicating that Otub1 increased Tau-seeded Tau aggregation in primary neurons, corroborating the results obtained in a nonneuronal cell line.

To measure the effect of Otub1 expression on oligomeric Tau forms, we performed Western blotting analysis in nonreducing conditions, following AAV-driven expression of Otub1 and GFP, in absence of Tau seeding. This revealed a significant increase of oligomeric Tau forms in AAV–Otub1-infected neurons compared with AAV–GFP-infected neurons (Fig. 4c). In addition, we found robust induction of a (homo- or heterotypic) Tau complex (Tau^c^), which was strongly detected in AAV–Otub1-infected neurons but absent in AAV–GFP-infected neurons, indicating clearcut Otub1-induced modulation of Tau in neurons. The induction of oligomeric Tau forms was further confirmed using dot-blot analysis with T22 antibody (Fig. 4d), revealing a significant increase in oligomeric Tau following expression of Otub1.

Taken together, our data demonstrate that Otub1, identified as a Tau-interacting protein in the Tau interactome mapping, modulates Tau, by increasing Tau-seeded Tau aggregation, by formation of a Otub1-induced Tau complex Tau^c^, and by increasing the concentration of oligomeric Tau forms in primary neurons.

### Otub1 but not Otub1-C91A impairs Tau degradation by removing Lys48-linked polyubiquitin chains from Tau in primary neurons

**Fig. 5:**
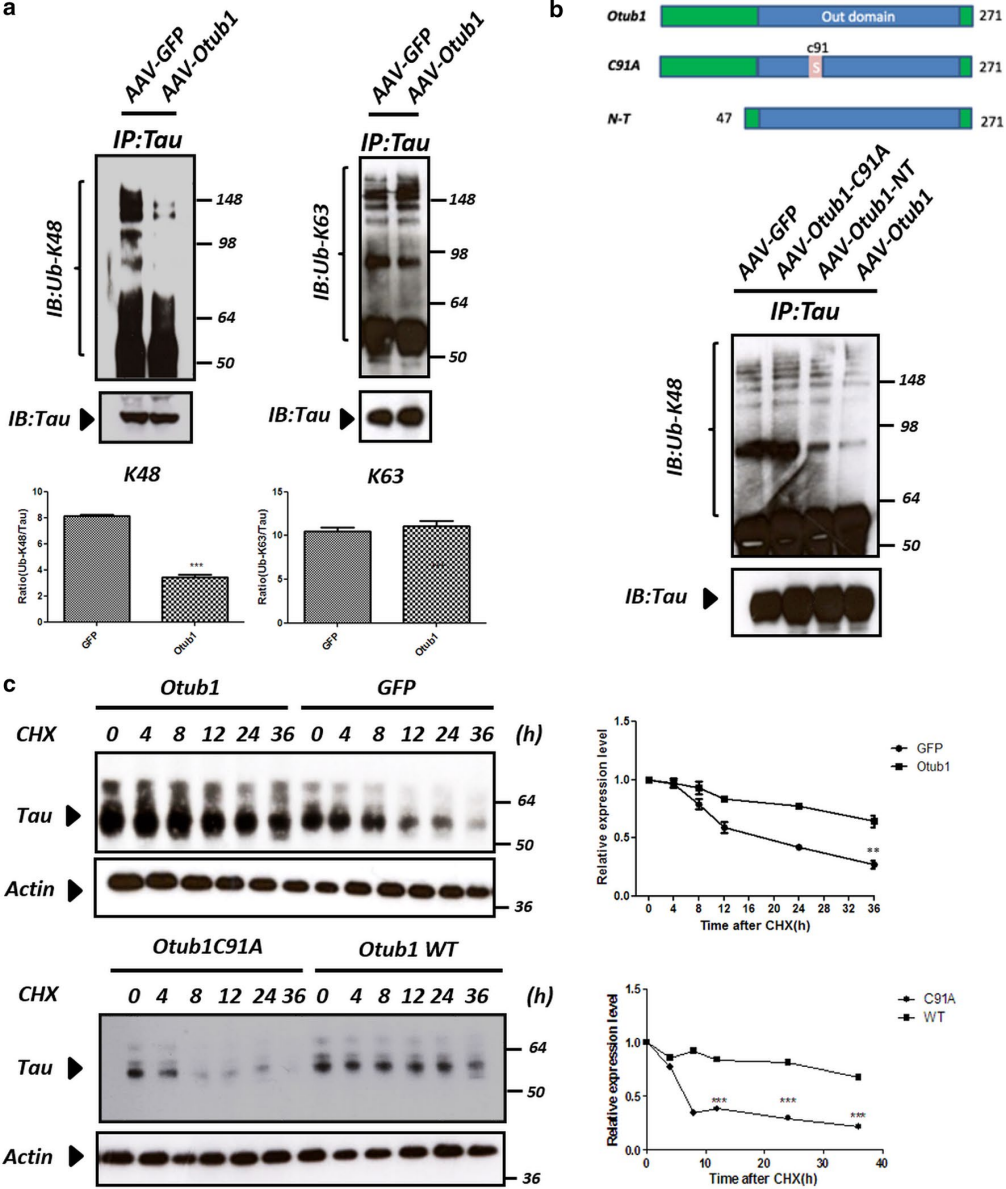
Otub1 but not Otub1-C91A increases Tau stability by removing Lys48-linked polyubiquitin chains from Tau in primary neurons. **a** Primary neurons from TauP301S mice were infected with AAV–Otub1 or AAV–GFP. At 7 days after infection, Tau proteins were immunoprecipitated with Tau antibody and analyzed by linkage-specific ubiquitin (Ub-Lys48, Ub-Lys63) immunoblotting. A representative blot is shown; quantification of three different experiments is presented (***p value <0.001). **b** Otub1-WT or catalytically dead mutant (C91A), N-terminal truncated mutant (N-T), and GFP control were expressed in primary TauP301S neurons. Immunoprecipitation was performed using Tau antibody, and Lys48-linked polyubiquitin chain formation was detected using Ub-Lys48 antibody. **c** Primary TauP301S neurons were infected with AAV–Otub1 compared with AAV–GFP. Five days later, neurons were treated with 10 µM protein synthesis inhibitor cycloheximide (CHX) for different durations up to 36 h as indicated. Similar comparison was performed for primary neurons from TauP301S mice infected with AAV–Otub1-WT compared with AAV–Otub1-C91A. The rate of Tau turnover was quantified using Western blot analysis. Actin served as loading control within each group. Quantification was performed on three independent experiments (**p value <0.01; ***p value <0.001)

Previous studies have shown that Otub1 has dual functions in regulating both ubiquitin assembly and disassembly [77, 78]. Otub1, as an isopeptidase, can remove Lys48-linked polyubiquitin chains from its substrates [17, 48, 74], but can also serve as an E2 enzyme inhibitor to impede Lys63-specific polyubiquitin chain formation in a noncanonical manner [61]. Since Otub1 interacts with Tau and promotes phosphorylated and aggregated Tau levels in primary neurons, we hypothesized that Otub1 could act as a Tau deubiquitinase, interfering with pathological Tau degradation and hence Tau aggregation. To test this assumption experimentally, we analyzed the ubiquitination status of Tau by immunoprecipitation. Following AAV-driven expression of Otub1 or GFP in primary neurons, Tau proteins were pulled down, and Lys48- and Lys63-linked polyubiquitination of Tau was measured by linkage-specific ubiquitin antibodies. To enrich ubiquitinated proteins, proteasome inhibitor was added to the medium 6 h before harvesting. Otub1 displayed strong preference for disassembling Lys48-linked polyubiquitin chains, while leaving Lys63-linked polyubiquitin chains unaffected (Fig. 5a). Analysis of a catalytically dead mutant with a C91A mutation revealed no effect on Lys48-linked polyubiquitination of Tau. Conversely, AAV-driven expression of N-terminally truncated Otub1 significantly decreased Lys48-linked polyubiquitin chains of Tau (Fig. 5b), demonstrating that the N-terminal domain of Otub1, which is crucial for its noncanonical role, is not involved in regulation of Tau ubiquitination.

To confirm the effect of Lys48-linked polyubiquitination change on Tau degradation, TauP301S neurons were infected with AAV–Otub1–GFP or AAV–GFP, and subsequently treated with protein synthesis inhibitor cycloheximide (CHX) for different durations up to 36 h to quantify the rate of Tau turnover using Western blot. We found that Tau degradation was significantly impaired in primary neurons infected with Otub1 WT, but not with catalytically dead mutant C91A, compared with GFP control. These results indicate that Otub1 regulates Tau degradation, dependent on its catalytic activity (Fig. 5c).

**Fig. 6:**
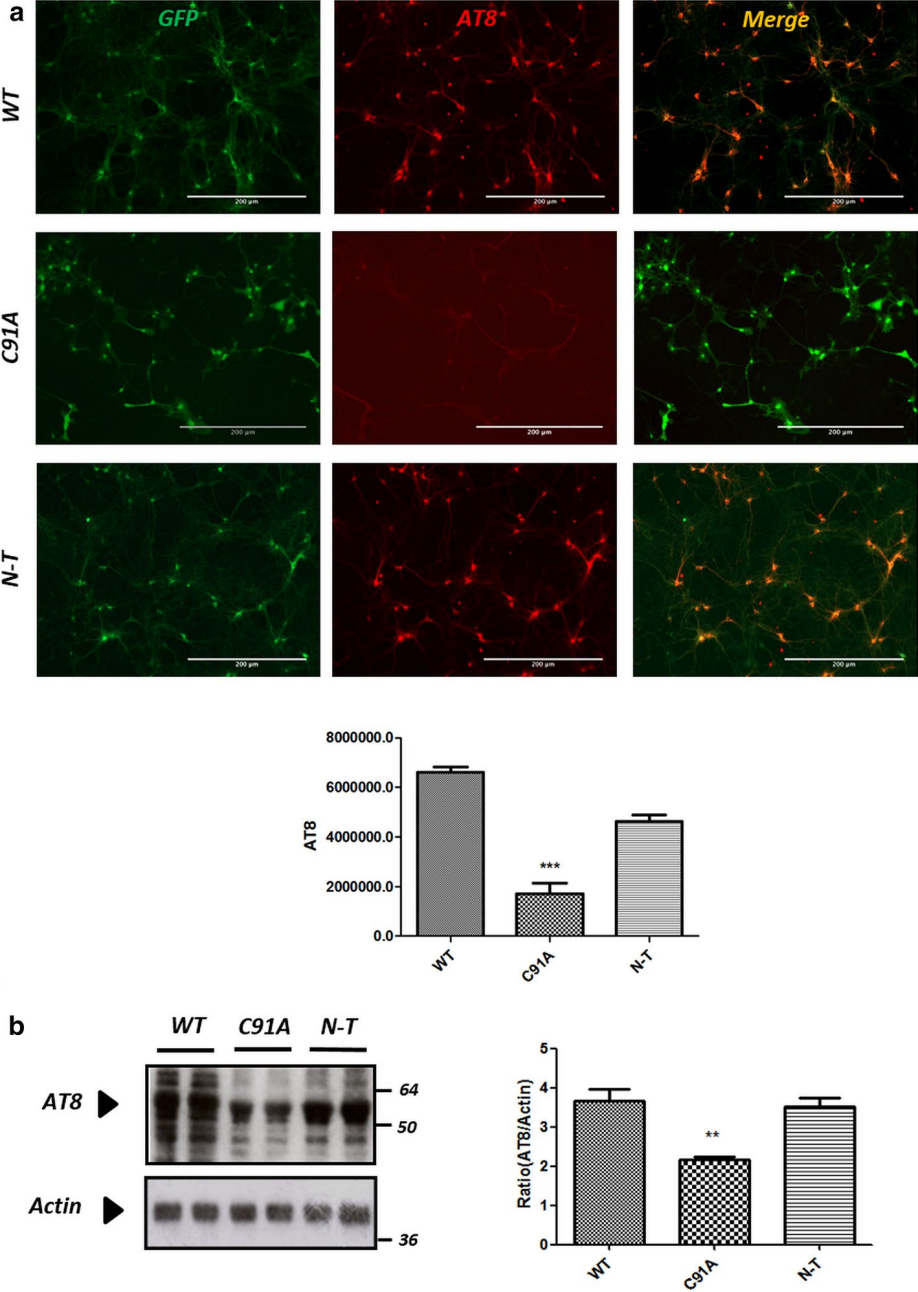
The effect of Otub1 on Tau phosphorylation is dependent on Otub1 catalytic activity. **a** Immunofluorescence staining of TauP301S primary neurons infected with AAV–Otub1 wild type (WT), catalytically dead mutant (C91A) or N-terminal truncation (N-T) using phospho-Tau antibody AT8 (Ser202/Thr205). Quantification of staining intensities is presented as mean ± SEM (***p value <0.01; *n* = 3 different infections; for each infection, three repetitions are analyzed). **b** Western blot analysis of AAV-infected primary neurons lysates using AT8 antibody. Actin was used as loading control (**p value <0.01; *n* = 3 different infections; for each infection, three repetitions were analyzed)

We further analyzed whether catalytically dead Otub1-C91A could affect Tau phosphorylation in primary neurons expressing TauP301S. AAV-driven expression of Otub1-C91A did not increase AT8-positive Tau, in contrast to expression of wild-type Otub1 and the N-terminally truncated form of Otub1. This was demonstrated by immunofluorescence staining (Fig. 6a) and confirmed by biochemical analysis (Fig. 6b).

Taken together, our data prove that Otub1 can remove Lys48-linked polyubiquitin chains from Tau in a catalytic activity-dependent manner, implicating the canonical pathway. Consequently, Otub1-dependent deubiquitination inhibits Tau degradation through the proteasome pathway and prolongs its half-life, leading to accumulation of pathological forms of Tau.

### Otub1 promotes Tau phosphorylation and oligomerization in vivo in Tau transgenic mice

**Fig. 7:**
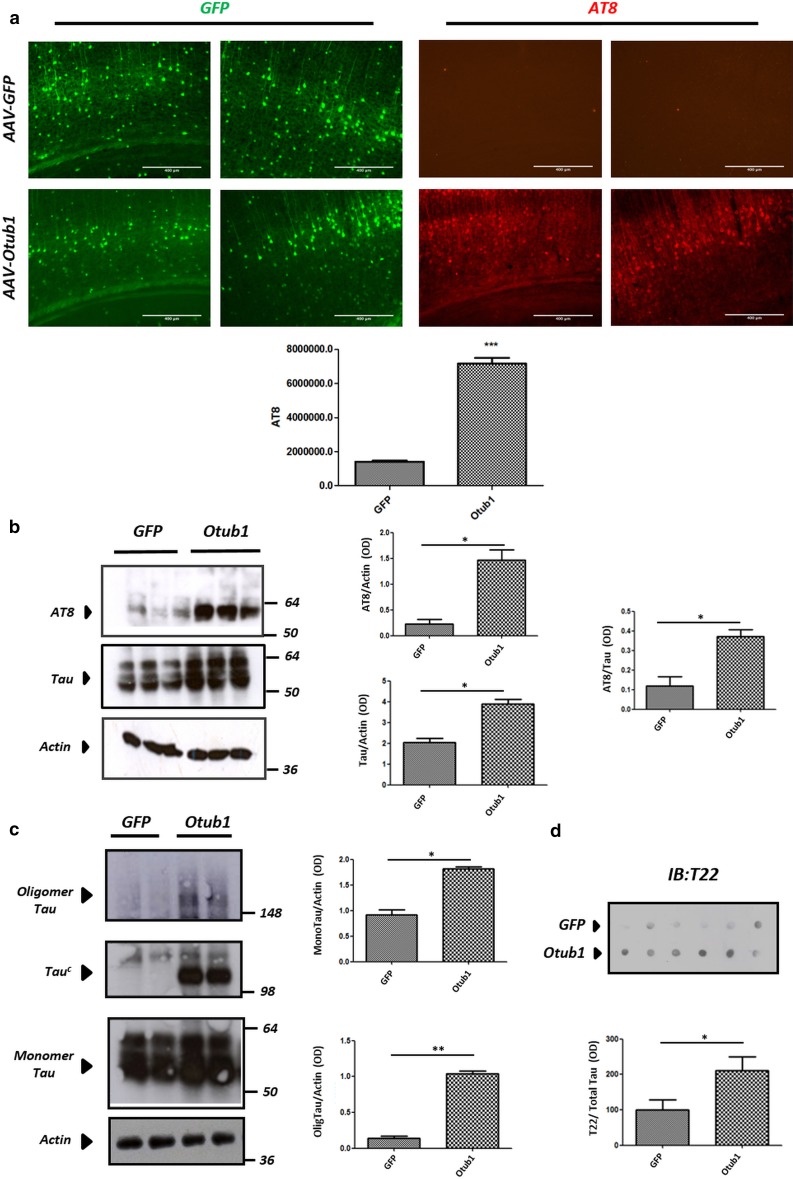
Otub1 increases concentrations of phosphorylated Tau and Tau oligomers in vivo in Tau transgenic mice. **a** Immunohistochemical staining of cortex of TauP301S transgenic mice injected with AAV–Otub1 or AAV–GFP at P0 and analyzed at 2 months. Representative images of AAV-driven expression of Otub1 and GFP are presented (*green*). Representative AT8 stainings are presented (*red*). Quantitative analysis is presented as mean ± SEM (*n* = 9 mice for each group; 3–5 sections were examined for each mouse; 9–12 fields were quantified for each section; *scale bar* 400 μm; ***p value <0.001). **b** Western blotting analysis of phosphorylated Tau and total Tau in cortex homogenates of AAV–Otub1- and AAV–GFP-injected mice, using AT8 (Ser202/Thr205) and anti-Tau antibody, is presented. Quantitation was performed on *n* = 9 mice for each group; the AT8/Tau ratio indicates the preferential effect of Otub1 on Tau phosphorylation (*p value <0.05). **c** Western blotting analysis of mice cortex homogenates in nonreducing condition reveals clear induction of oligomeric forms of Tau (detected as high-MW smears), as well as monomeric Tau protein level increase in TauP301S mice injected with AAV–Otub1 (*n* = 9 mice for each group; *p value <0.05; **p value <0.01). Induction of a Tau complex (homo- or heteromeric) of ~120 kDa—denoted Tau^c^—is clearly detected following AAV–Otub1 but not AAV–GFP infection. **d** Dot-blot analysis of mice cortex homogenates using oligomeric Tau-specific antibody T22 is presented. Quantitative analysis was performed following normalization to total Tau (dot-blot analysis) (*n* = 6 mice for each group; *p value <0.05)

To investigate the effect of Otub1 on Tau in vivo, AAV–Otub1 and AAV–GFP were bilaterally intraventricularly injected into TauP301S transgenic pups (P0), resulting in widespread expression of Otub1 and GFP in cortical neurons at 2 months of age (Fig. 7a). Immunostaining with AT8 antibody revealed abundant AT8-positive neurons in AAV–Otub1-injected mice, absent in AAV–GFP-infected mice (Fig. 7a), at 2 months postinjection. Biochemical analysis further confirmed increased AT8-positive Tau by Otub1 expression (Fig. 7b), with AT8 phosphorylated Tau more robustly increased than total Tau, as reflected by the AT8/total Tau ratio. Cortex homogenates from TauP301S transgenic mice injected with AAV–Otub1 and control mice injected with AAV–GFP were analyzed under nonreducing condition. This revealed a significant increase of monomeric Tau and oligomeric Tau in AAV–Otub1-injected mice compared with AAV–GFP-injected mice (Fig. 7c). Similarly as in primary neurons, Tau^c^ (a homo- or heterotypic Tau complex) was robustly induced in AAV–Otub1- but not AAV–GFP-injected mice, strongly confirming Otub1-induced modulation of Tau in brain (Fig. 7c). To further confirm the increased concentration of oligomeric Tau forms following Otub1 expression, dot-blot analysis using oligomeric Tau-specific antibody T22 was performed. This revealed significantly increased oligomeric Tau in AAV–Otub1-injected mice compared with AAV–GFP-injected control cases (Fig. 7d).

### The effect of Otub1 on Tau phosphorylation and oligomerization in vivo is dependent on its catalytic function

**Fig. 8:**
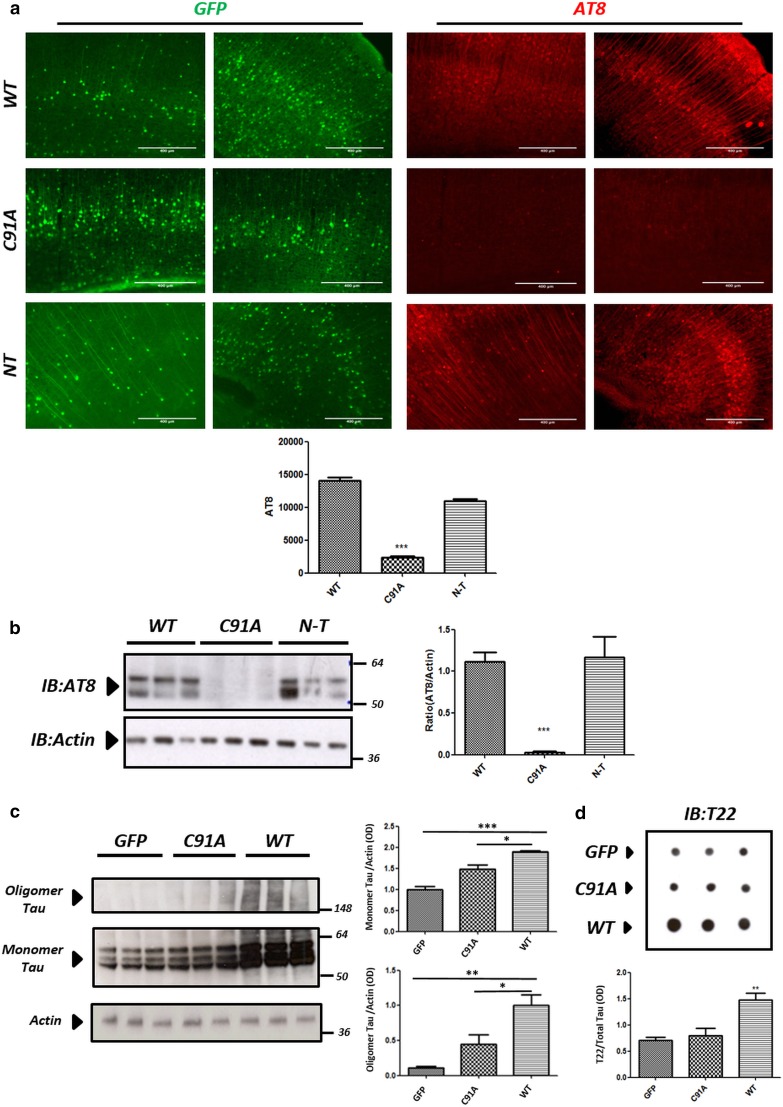
The effect of Otub1 on Tau phosphorylation and oligomerization in vivo is dependent on its catalytic function. **a** Immunohistochemical staining of cortex of TauP301S transgenic mice injected with AAV–Otub1 (WT), catalytically dead mutant (C91A) or N-terminal truncation (N-T) at P0 and analyzed at 2 months. Representative images of AAV-driven expression of Otub1 (wild type, C91A, NT) are presented (*green*). Representative AT8 stainings are presented (*red*) for each group (3–5 sections were examined for each mouse; 9–12 fields were quantified for each section; scale bar 400 μm, ***p value <0.001). **b** Western blotting analysis was used to analyze phosphorylated Tau in cortex homogenates of mice injected with AAV–Otub1 wild type or two mutants, AAV–Otub1 C91A and AAV–Otub1 N-T, using AT8 (Ser202/Thr205) antibody (*n* = 3 mice for each group; ***p value <0.001). **c** Western blotting analysis of cortex homogenates from TauP301S mice injected with AAV–Otub1 wild type, with AAV–Otub1 C91A, or AAV–GFP in nonreducing condition using Tau antibody is presented. This reveals a clear increase in oligomeric forms of Tau only in TauP301S mice injected with AAV–Otub1 wild type, while not in AAV–Otub1 C91A- or AAV–GFP-injected TauP301S mice (n = 3 mice for each group; *p value <0.05; **p value <0.01). Monomeric Tau increased, less strongly compared with oligomeric Tau, in TauP301S mice injected with AAV–Otub1 wild type, compared with AAV–Otub1 C91A- or AAV–GFP-injected TauP301S mice (*n* = 3 mice for each group; *p value <0.05; ***p value <0.001). **d** Dot-blot analysis of cortex homogenates from TauP301S mice injected with AAV–Otub1 wild type, with AAV–Otub1 C91A, or AAV–GFP using oligomeric Tau-specific antibody T22 is presented. Quantitative analysis was performed following normalization to total Tau (dot-blot analysis) (*n* = 3 mice for each group; **p value <0.01)

To further assess the requirement for catalytically active Otub1 and the presence of the catalytically conserved C91 of Otub1, for accumulation of pathological Tau forms in vivo, we performed P0 injections of AAV–Otub1-C91A or AAV–Otub1-N-terminal truncated mutant, and AAV–Otub1-WT in TauP301S mice. Immunostaining and biochemical analysis, 2 months postinjection, unambiguously revealed that the catalytically dead mutant C91A did not affect AT8-stained Tau in vivo, while N-terminal truncated mutant of Otub1 increased AT8-positive Tau in vivo, similarly as wild-type Otub1 (Fig. 8).

To further analyze the requirement for catalytically active Otub1 for induction of oligomeric Tau forms, brain extracts of AAV-infected TauP301S mice were biochemically analyzed 2 months postinjection, under nonreducing condition. This revealed significantly increased oligomeric Tau forms, following expression of wild-type Otub1, but not following expression of GFP or of the catalytically inactive form of Otub1 (C91A) (Fig. 8c). This was further confirmed by dot-blot analysis using T22 antibody recognizing oligomeric Tau forms (Fig. 8d). These data indicate that expression of catalytically active Otub1 is required for accumulation of phosphorylated Tau and oligomeric Tau forms.

Noteworthy, to analyze whether endogenous Otub1 is modulated in conditions of accumulating pathological Tau forms, we took advantage of our microarray analysis performed for a different project in which we analyzed time-dependent changes in gene expression in hippocampus following Tau-seeded Tau aggregation in Tau transgenic mice. Interestingly, Otub1 expression is gradually downregulated with increasing Tau-seeded Tau aggregation (Fig. S4a), which could be considered as a protective mechanism. Furthermore, analysis of hippocampal gene expression in aging mice, using publicly available data (NCBI database GDS2082) [73], indicates a significant increase in Otub1 expression with aging (Fig. S4b). The latter suggests that increased expression of Otub1 with aging might contribute to increased risk for accumulation of pathological Tau forms with aging.

## Discussion

Accumulation of aberrantly folded proteins is common to many neurodegenerative disorders, including AD, making (dys)regulation of proteostasis not only a potentially implicated pathogenetic mechanism, but most importantly an attractive therapeutic target. In AD, symptom progression strongly correlates with progression of Tau pathology, with early, soluble, oligomeric forms of Tau generally considered as toxic culprits. Aberrant protein degradation occurs through either the ALS or UPS, with the former mainly involved in degradation of large insoluble protein aggregates, while the latter is preferentially involved in clearance of smaller soluble forms, being the focus of this work. In view of the importance of the UPS in AD, Tau-ubiquitinating enzymes have been identified, while Tau deubiquitination has received less attention. Starting from a Tau interactome mapping, we identified here for the first time Otub1 as a novel Tau deubiquitinase, affecting accumulation of pathological forms of Tau in vitro and in vivo.

Here, we present a proteome-wide screening approach to identify Tau-interacting proteins, as a basis to gain insight into Tau pathophysiology and to identify Tau modifiers with therapeutic potential. Proteome-wide screening approaches are increasingly used—in analogy to genome-wide screening approaches—to gain novel insights from an unbiased starting point for drug discovery and fundamental science [3, 10, 27, 58, 62]. We used an iTRAQ-based approach to identify proteins interacting with endogenous Tau from mouse brain. The validity of our approach is reflected in the identification of several well-characterized Tau-interacting proteins and the current identification of Otub1 as a Tau deubiquitinase. The presented Tau interactome provides important information, extending beyond the current work, providing the basis for novel insights into the pathological role of Tau and to identify novel Tau-directed targets.

Here, we validated this approach and identified for the first time Otub1 as a novel Tau deubiquitinase, based on the Tau interactome map. Accumulating evidence implicates dysregulated proteostasis and a dysregulated UPS in AD, as highlighted in the “Introduction.” Different types of polyubiquitination of Tau, including Lys48 and Lys63 linked, have been identified in AD patients by mass spectrometry and immunological analysis [13, 49–51, 56]. Interestingly, we here identified Otub1 as a Tau deubiquitinase which decreases the levels of Lys48- but not Lys63-linked polyubiquitin chains on Tau, increasing Tau stability in neurons. Our finding is in line with previous reports showing that Otub1 has preference for Lys48-linked polyubiquitination [17, 48, 74] and functions as a regulator of protein turnover [23, 26, 32, 44, 66]. Mutation of the catalytically conserved cysteine (C91) abolished its role in Tau polyubiquitination, further confirming the crucial role of its catalytic capacity for its involvement in the regulation of Lys48 polyubiquitination on Tau. Otub1 has also been found to have a noncanonical role in DNA damage response by impeding Lys63 polyubiquitin chain formation [53, 77, 78], but no effect of Otub1 on Lys63 polyubiquitination of Tau nor implication of the N-terminal part of Otub1 was demonstrated.

Notably, the effect of polyubiquitination modification is determinant for the fate of its substrate. Lys48 polyubiquitin chains act as a proteasomal degradative signal, while Lys63-linked ubiquitin modification has been proposed to contribute to biogenesis of inclusions and clearance of larger aggregates by autophagy [36, 55, 68, 80]. Although soluble Tau forms as well as large insoluble aggregated Tau forms can be polyubiquitinated [13, 49, 50], higher-order protein aggregations are unlikely to pass through the narrow proteasome opening, and they are normally sequestrated into inclusions or aggresomes and cleared through the ALS. Recently, several lines of evidence have indicated selective autophagy using Lys63-linked polyubiquitin chains as a cargo recognition signal [36, 55, 68, 80]. In our experiments, we found that Otub1 only influences Lys48 polyubiquitination but not Lys63-linked polyubiquitin chains of Tau, and therefore would be rather involved in clearance of smaller soluble forms of Tau through the proteasome.

These findings are further strengthened by our assessment of the effect of Otub1 on Tau metabolism. We demonstrate that Otub1-dependent Tau deubiquitination is linked to accumulation of Tau phosphorylated at the pathologically relevant AT8 epitope—used for Braak staging—and accumulation of oligomeric soluble forms of Tau, which have been considered to be toxic culprits. Hence, overexpressed Otub1, which regulates Lys48-linked polyubiquitination of Tau, promotes accumulation of abnormally phosphorylated Tau and oligomeric Tau, both in Tau transgenic mice and in primary neurons. These findings are in line with previously published findings. Depletion of CHIP in Tau mice leads to significant accumulation of ubiquitin-negative, and phosphorylated soluble Tau species [16]. In a nonneuronal cell line which overexpresses the longest human Tau isoform, proteasome inhibitor treatment stabilizes phosphorylated and aggregated Tau species which arise from Tau phosphatase PPA inactivation and normally decay within 24 h [21]. In a triple transgenic mouse model, amyloid β-peptide (Aβ) immunotherapy leads to clearance of early soluble Tau forms by the UPS, but not of late Tau aggregates [54]. Along the same vein, molecular chaperon FKBP51, together with Hsp90, increase Tau oligomer formation by inhibiting Tau degradation through the proteasome in a mouse model [8]. In line with these reports, our work corroborates regulation of the early pathological Tau forms by the UPS, including deubiquitination as an important modulator.

Our data are important in view of the fact that early oligomeric forms of Tau are generally considered to be toxic culprits in AD and related Tauopathies. We have previously shown in a Tau seeding assay in in vitro and in vivo models that neuronal dysfunction correlated strongly with early pathological forms of Tau rather than with full-blown mature NFTs [65]. Previous studies using elegant approaches have indicated that the Tau aggregation process and early pathological forms rather than full-blown NFTs correlate with neuronal dysfunction [6, 11, 12, 15, 33, 37, 39, 40, 59, 60, 67]. The detrimental role of Tau oligomers in neuronal function and behavior was further demonstrated using acute injection of oligomeric Tau and immunization with anti-Tau oligomeric antibodies [11, 12, 39, 40]. Taken together, these data highlight the importance of targeting early oligomeric Tau forms as pathogenetic culprits, and hence the importance of the demonstrated effect of Otub1 on soluble oligomeric forms of Tau.

Interestingly, we show that the conserved catalytic cysteine (C91) of Otub1 is crucial for accumulation of hyperphosphorylated Tau and oligomeric Tau species. This finding is not only important as an internal control but also in the context of drug development, providing a druggable target. It must be noted that upstream modulators of Otub1, mechanisms regulating Otub1 activity, expression and probably the binding of Otub1 to chaperone molecules must be considered as potential therapeutic targets as well. Regulation of key proteins via recruitment of distinct E3 ligases or DUBs mediated by specific adaptor proteins is common in many cellular pathways. While the mechanisms upstream of Otub1 remain unknown, they may provide additional therapeutic targets. Inhibition of deubiquitinases as therapeutic targets is attracting increasing attention for different diseases, including cancer. Our findings identifying Otub1 as a Tau deubiquitinase affecting pathological forms of Tau in vitro and in vivo may therefore yield new perspectives for therapy.

Taken together, in this work we present a Tau interactome map, which yields a basis for novel insights into Tau biology and its pathological role, validated by and extending beyond our current work. We furthermore provide novel insight into the link between the UPS and AD and open new avenues for development of small-molecule inhibitors specifically targeting accumulation of pathological forms of Tau in AD and related Tauopathies.

## Electronic supplementary material

Below is the link to the electronic supplementary material.

Supplementary material 1 (DOCX 35 kb)

Supplementary material 2 (DOCX 19 kb)

Supplementary material 3 (TIFF 54583 kb)

Supplementary material 4 (XLSX 83 kb)

Supplementary material 5 (XLSX 167 kb)

Supplementary material 6 (XLSX 25 kb)

Supplementary material 7 (XLSX 24 kb)

Supplementary material 8 (TIFF 50617 kb)

Supplementary material 9 (TIFF 34212 kb)

Supplementary material 10 (TIFF 22053 kb)
