# Supplementary material for: Tau interactome mapping based identification of Otub1 as Tau deubiquitinase involved in accumulation of pathological Tau forms in vitro and in vivo
Source: Acta Neuropathol. 2017 Jan 12;133(5):731–49. doi: 10.1007/s00401-016-1663-9 (PMC5390007; doi:10.1007/s00401-016-1663-9)
Supplement: Supplementary file 1 — Supplementary material 1 (DOCX 35 kb) [file 401_2016_1663_MOESM1_ESM.docx]

**Tau-interactome mapping based identification of Otub1 as Tau-deubiquitinase, involved in accumulation of pathological Tau forms in vitro and in vivo.**

**Acta neuropathologica**

Peng Wang^1^, Gerard Joberty^3^, Arjan Buist^2^, Alexandre Vanoosthuyse^1^, Ilie-Cosmin Stancu^1^, Bruno Vasconcelos^1^, Nathalie Pierrot^1^, Maria Faelth-Savitski^3^, Pascal Kienlen-Campard^1^, Jean-Noël Octave^1^, Marcus Bantscheff^3^, Gerard Drewes^3^, Diederik Moechars^2^, Ilse Dewachter^1,4#^

1. Alzheimer Dementia Group, Institute of Neuroscience, Catholic University of Louvain, 1200 Brussels, Belgium

2. Department of Neuroscience, Janssen Research and Development, A Division of Janssen Pharmaceutica NV, 2340 Beerse, Belgium

3. Cellzome GmbH, Molecular Discovery Research, GlaxoSmithKline, Meyerhofstrasse 1, Heidelberg, Germany

4. BioMedical Research Institute, UHasselt, Belgium

# Corresponding author: ilse.dewachter@uclouvain.be

**Supplemental Experimental Procedures**

**Tau-interactome mapping**

*Sample preparation and co-immuneprecipitation.* Mouse brains were cut into pieces and homogenized in lysis buffer (50 mM Tris, pH 7.4, 5 % glycerol, 1.5 mM MgCl2, 100 mM NaCl, 25 mM NaF, 1 mM Na3VO4) using a Polytron. NP-40 detergent was added to 0.8% as well as DNAseI. Sample was further homogenized using a douncer. After 30 min incubation, lysate was spun 10 min at 20,000 g. Supernatant was then diluted 2 fold in lysis buffer without detergent and spun 1 h at 100,000 g. Anti-Tau antibodies were cross-linked to Sepharose beads using NaCNBH3 according to the manufacturer’s instruction. For each immunoprecipitation, 10 mg of brain lysate was used with 100 µL of antibody beads. Immunoprecipitates were washed with lysis buffer with 0.4% NP-40 and then with lysis buffer without detergent. Proteins were alkylated with 200 mg/ml iodoacetamide, separated on 4–12% NuPAGE gels (Invitrogen), and stained with colloidal Coomassie.

*Mass Spectrometry.* Procedures were essentially as previously reported [2]. Gels were cut into slices across the entire separation range and subjected to in-gel digestion. Peptide extracts were labeled with iTRAQ™ (Applied Biosystems). Samples from each immunoprecipitation were labelled with iTRAQ reagents, combined, dried and acidified prior to LC-MS/MS analysis using 2 hr gradient separation on a nano-flow HPLC system (Eksigent, USA). Fragment spectra were generated using Pulsed-Q dissociation, as described [1]. Raw data were processed into mgf files using an in-house developed software. Mgf files were searched using Mascot (2.2) with carbamidomethyl cysteine and Lysine iTRAQ as fixed and oxidized methionine, acetylated protein N-terminus, and N-terminal iTRAQ as variable modifications. Trypsin was specified as the proteolytic enzyme and up to three missed cleavages were allowed. The mass tolerance of precursor ions was set to 3 Da and that of fragment ions was set to 0.6 Da. The data was searched against an in-house curated version of the human International Protein Index database combined with a decoy version thereof and supplemented with a set of common contaminant proteins. Protein identification acceptance criteria were based on spectrum to peptide sequence assignments that had a 10x higher Mascot probability than the second best match. For protein identifications with only one single peptide meeting these criteria, we required the Mascot score to be at least 50. Thus, proteins were identified with FDR < 0.1%. Quantification was performed only on proteins with at least four spectrums to sequence assignments. No decoy data base identifications were observed for quantified proteins.

*Statistical analysis of data.* A variance stabilizing transformation was applied using a parameterized arsinh transformation to obtain normal distribution of noise [3, 5]. Protein fold changes vs. control antibodies were calculated by fitting weighted Generalized Additive Mixed Models (GAMMs) via the R-package gam. The following influence factors were considered in the GAMMs: protein, antibody, label and experiment specific factors. One-sample t-tests were employed for assessing the significance of protein enrichment. Correction for multiple testing was then performed using Benjamini and Hochberg's FDR method [6]. Gene ontology and KEGG pathway enrichment analysis of the proteins identified as Tau interactors was done using DAVID (version 6.8) [4] using the whole mouse genome as a background. Gene ontology terms and KEGG pathway with false discovery rate (FDR) < 0.05 was considered significant.

The protein-protein interaction database STRING [8, 10] was used to visualize known protein-protein interactions between the proteins identified as Tau interactors. For the visualization the following parameters were used: active interaction source: Experiments and Databases, minimum required interaction score: high confidence (0.700)

**Micro-array analysis of Tau-seeded Tau transgenic mice**

Tau transgenic mice, expressing the longest human tau isoform with the P301L mutation (Tau-4R/2N-P301L) under control of the thy1 gene promoter were used [11]. The animals were used for surgery at the age of 3 months, which is at 6 months before they develop tau pathology driven solely by the Tau transgene. All experiments were performed in compliance with protocols approved by the local ethical committee. Tau P301L mice were seeded with Tau-seeds as described previously [7, 9]. Three months old Tau P301L mice, were either non-injected, injected with buffer or injected with Tau-seeds, and analyzed either 2 or 4 weeks post-injection. For micro-array analysis, total RNA was isolated from the CA1 region that was dissected from transversally cut 400 µm thick sections around the injection coordinates using the RNeasy kit (Qiagen, Venlo, Netherlands) combined with DNAse treatment. One μg of extracted total RNA was biotin labeled and hybridized with the Mouse Genome 430 version 2.0 GeneChip array (Affymetrix, Orbassano, Italy). All data were processed by using the statistical computing R-program (Rversion 2.4.0) and Bioconductor tools. The computed gene expressions were computed using the FARMS algorithm. Grouping of the individual probes into gene-specific probe sets was performed based on the Entrez. Analysis of the microarray data were done looking at significance analysis of microarray (SAM, at FDR < 0.005).

**References**

1. Bantscheff M, Boesche M, Eberhard D, Matthieson T, Sweetman G, Kuster B (2008) Robust and sensitive iTRAQ quantification on an LTQ Orbitrap mass spectrometer. Mol Cell Proteomics 7: 1702-1713. doi 10.1074/mcp.M800029-MCP200

2. Bantscheff M, Hopf C, Savitski MM, Dittmann A, Grandi P, Michon AM, Schlegl J, Abraham Y, Becher I, Bergamini Get al (2011) Chemoproteomics profiling of HDAC inhibitors reveals selective targeting of HDAC complexes. Nat Biotechnol 29: 255-265. doi 10.1038/nbt.1759

3. Ennis M, Hinton G, Naylor D, Revow M, Tibshirani R (1998) A comparison of statistical learning methods on the Gusto database. Stat Med 17: 2501-2508

4. Huang da W, Sherman BT, Lempicki RA (2009) Systematic and integrative analysis of large gene lists using DAVID bioinformatics resources. Nat Protoc 4: 44-57. doi 10.1038/nprot.2008.211

5. Huber W, von Heydebreck A, Sultmann H, Poustka A, Vingron M (2002) Variance stabilization applied to microarray data calibration and to the quantification of differential expression. Bioinformatics 18 Suppl 1: S96-104

6. Klipper-Aurbach Y, Wasserman M, Braunspiegel-Weintrob N, Borstein D, Peleg S, Assa S, Karp M, Benjamini Y, Hochberg Y, Laron Z (1995) Mathematical formulae for the prediction of the residual beta cell function during the first two years of disease in children and adolescents with insulin-dependent diabetes mellitus. Med Hypotheses 45: 486-490

7. Peeraer E, Bottelbergs A, Van Kolen K, Stancu IC, Vasconcelos B, Mahieu M, Duytschaever H, Ver Donck L, Torremans A, Sluydts Eet al (2015) Intracerebral injection of preformed synthetic tau fibrils initiates widespread tauopathy and neuronal loss in the brains of tau transgenic mice. Neurobiol Dis 73: 83-95. doi 10.1016/j.nbd.2014.08.032

8. Snel B, Lehmann G, Bork P, Huynen MA (2000) STRING: a web-server to retrieve and display the repeatedly occurring neighbourhood of a gene. Nucleic Acids Res 28: 3442-3444

9. Stancu IC, Vasconcelos B, Ris L, Wang P, Villers A, Peeraer E, Buist A, Terwel D, Baatsen P, Oyelami Tet al (2015) Templated misfolding of Tau by prion-like seeding along neuronal connections impairs neuronal network function and associated behavioral outcomes in Tau transgenic mice. Acta Neuropathol 129: 875-894. doi 10.1007/s00401-015-1413-4

10. Szklarczyk D, Franceschini A, Wyder S, Forslund K, Heller D, Huerta-Cepas J, Simonovic M, Roth A, Santos A, Tsafou KPet al (2015) STRING v10: protein-protein interaction networks, integrated over the tree of life. Nucleic Acids Res 43: D447-452. doi 10.1093/nar/gku1003

11. Terwel D, Lasrado R, Snauwaert J, Vandeweert E, Van Haesendonck C, Borghgraef P, Van Leuven F (2005) Changed conformation of mutant Tau-P301L underlies the moribund tauopathy, absent in progressive, nonlethal axonopathy of Tau-4R/2N transgenic mice. J Biol Chem 280: 3963-3973. doi 10.1074/jbc.M409876200
