# Supplementary material for: Tau interactome mapping based identification of Otub1 as Tau deubiquitinase involved in accumulation of pathological Tau forms in vitro and in vivo
Source: Acta Neuropathol. 2017 Jan 12;133(5):731–49. doi: 10.1007/s00401-016-1663-9 (PMC5390007; doi:10.1007/s00401-016-1663-9)
Supplement: Supplementary file 2 — Supplementary material 2 (DOCX 19 kb) [file 401_2016_1663_MOESM2_ESM.docx]

**Tau-interactome mapping based identification of Otub1 as Tau-deubiquitinase, involved in accumulation of pathological Tau forms in vitro and in vivo.**

**Acta neuropathologica**

Peng Wang^1^, Gerard Joberty^3^, Arjan Buist^2^, Alexandre Vanoosthuyse^1^, Ilie-Cosmin Stancu^1^, Bruno Vasconcelos^1^, Nathalie Pierrot^1^, Maria Faelth-Savitski^3^, Pascal Kienlen-Campard^1^, Jean-Noël Octave^1^, Marcus Bantscheff^3^, Gerard Drewes^3^, Diederik Moechars^2^, Ilse Dewachter^1,4#^

1. Alzheimer Dementia Group, Institute of Neuroscience, Catholic University of Louvain, 1200 Brussels, Belgium

2. Department of Neuroscience, Janssen Research and Development, A Division of Janssen Pharmaceutica NV, 2340 Beerse, Belgium

3. Cellzome GmbH, Molecular Discovery Research, GlaxoSmithKline, Meyerhofstrasse 1, Heidelberg, Germany

4. BioMedical Research Institute, UHasselt, Belgium

# Corresponding author: ilse.dewachter@uclouvain.be

**Legends of supplemental figures and tables**

**Fig. S1 STRING analysis of Tau-interacting proteins**

STRING analysis was performed on the identified Tau-interacting proteins highlighting known interactions between the identified proteins.

**Table S1**

Complete list of all proteins significantly enriched with at least one of the 3 anti-Tau antibody when compared to control immunoprecipitations. Enrichment values (as log2) and P-values are displayed.

**Table S2**

Complete list of all proteins identified in each experiment. Sum ion area and enrichment (relative to control immunoprecipitation) are displayed.

**Table S3**

David Gene ontology and KEGG pathway enrichment analysis of the proteins identified as Tau interactors. Gene ontology categories are: Biological process, Cellular compartment and Molecular function. All results with FDR <0.05 are displayed.

**Table S4 Complete Tau-interactome map: Full list of Tau interacting proteins grouped by function.**

Full list of Tau interacting proteins grouped by function. This clustering is based on Gene ontology and was used to generate **Figure 1c**.

**Fig. S2 Cytological and biochemical characterization of the cellular Tau-seeded Tau-aggregation model**

**a.** QBI293 cells, stably expressing the longest human Tau isoform (2N4R) with aggregation-prone P301L mutation and GFP tag, were seeded with preformed pre-aggregated synthetic Tau seeds to induce monomeric Tau aggregation. Robust aggregation was assessed by stringent extraction using 1% Triton X100, removing soluble proteins. AT8 staining was used to monitor aggregated Tau forms. Clearcut overlap between GFP signal and AT8 signal validated this assay. **b.** QBI293 cells, seeded with pre-aggregated Tau seeds, were subject to sequential extraction, first by Triton containing lysis buffer, followed by SDS lysis buffer. Tau and AT8 antibody were respectively used to detect protein level of total Tau and phosphorylated Tau in soluble and insoluble fractions.

**Fig. S3 Selective effect of Otub1 on Tau aggregation in a well-characterized cellular Tau aggregation model**

**a.** Different Dubs plasmids (with flag tag) were transfected into QBI-293 cells one day before seeding, and cells were seeded for three days before analysis. **b.** Quantification reveals significant increase of Tau aggregation following Otub1 expression but not in other Dubs transfected cells (n>36 fields per condition; **p value <0.01; ANOVA with posthoc Bonferroni analysis).

**Fig. S4 Quantitative analysis of Otub1 expression using micro-array analysis is presented**

**a.** Micro-array analysis was performed on hippocampus of Tau-seeded mice and buffer injected TauP301L mice and analyzed at 1 or 3 months post-seeding. We have previously shown that Tau-seeding induces robust Tau-aggregation and accumulation of early soluble pathological Tau-forms [74]. A significant decrease in Otub1 expression is induced at 2 weeks post Tau-seeding, and becomes more pronounced at 4 weeks post-Tau-seeding (n=8 samples per condition, p-values *<0,05; ***<0,001; ANOVA with posthoc Bonferroni analysis). **b.** Existing data from a publically available data-base were used to analyze the effect of aging on Otub1 expression in hippocampus (NCBI database GDS2082). This revealed a significant increase of Otub1 expression in 15 months old non transgenic mice compared to 2 months old non-transgenic mice [n=9 (2mo); n=14 (15mo); p-value *<0,05].
