# Supplementary figures and images for: Tau interactome mapping based identification of Otub1 as Tau deubiquitinase involved in accumulation of pathological Tau forms in vitro and in vivo
Source: Acta Neuropathol. 2017 Jan 12;133(5):731–49. doi: 10.1007/s00401-016-1663-9 (PMC5390007; doi:10.1007/s00401-016-1663-9)

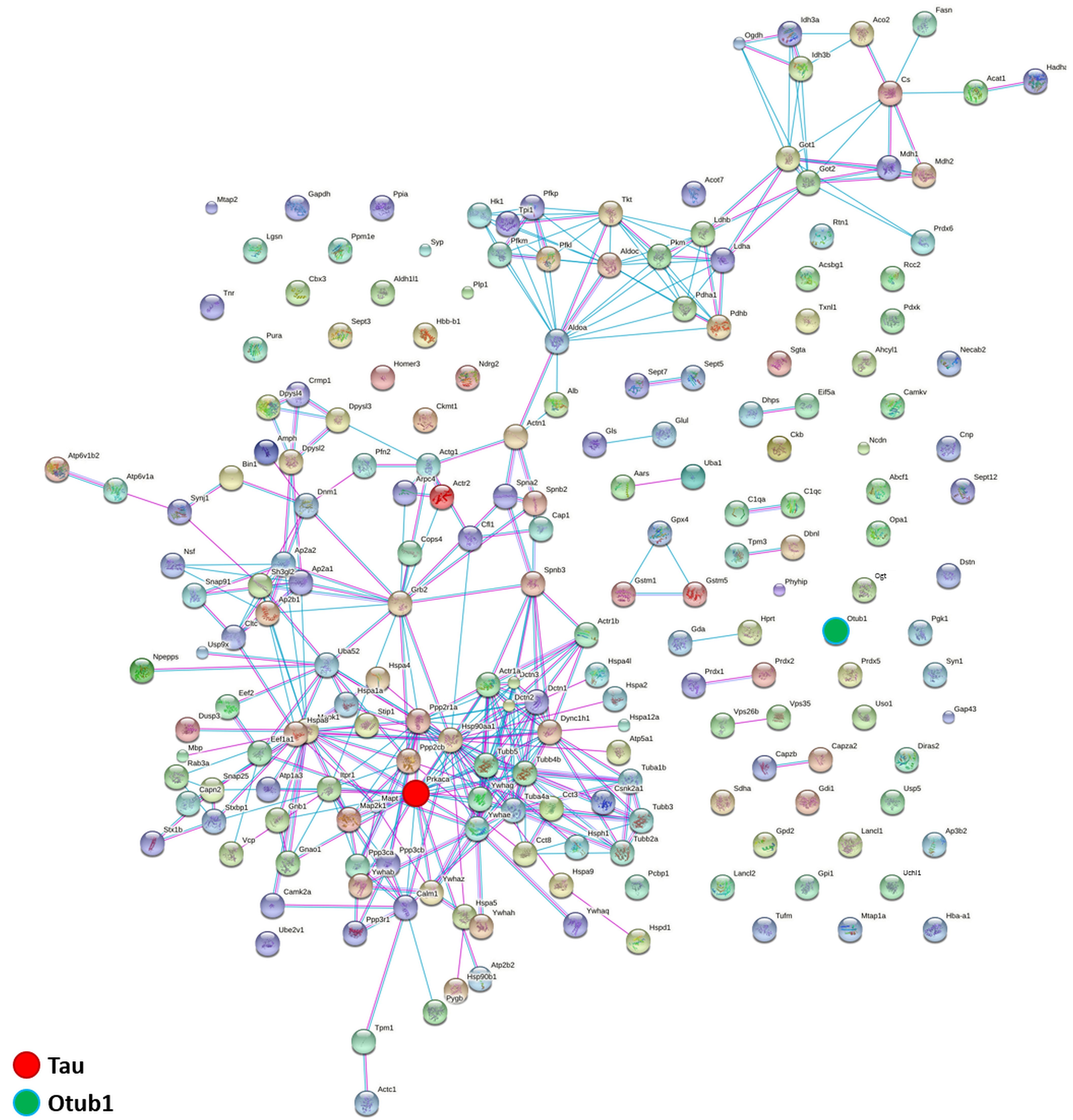

Supplement: Supplementary file 3 — Supplementary material 3 (TIFF 54583 kb) [file 401_2016_1663_MOESM3_ESM.tif]

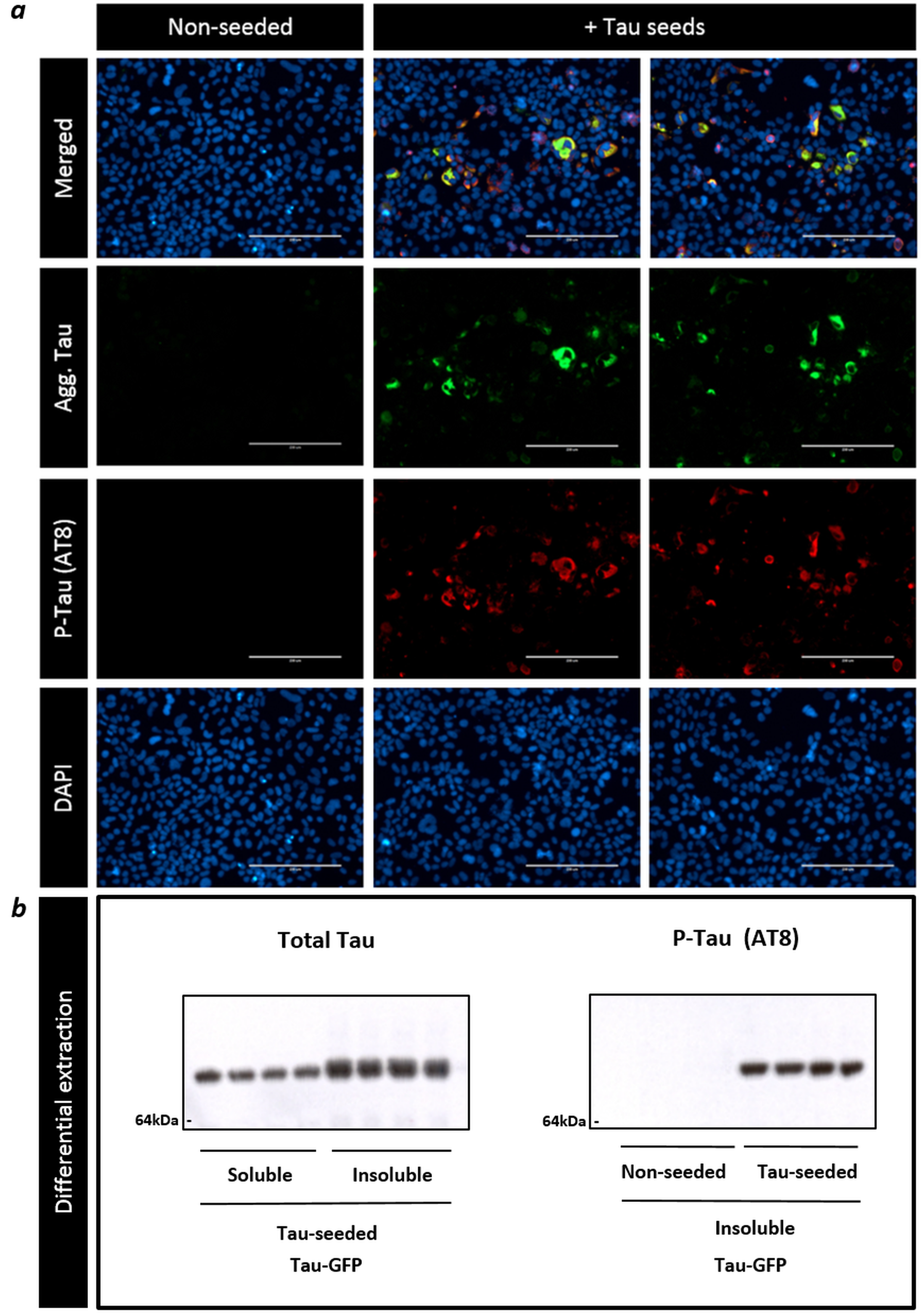

Supplement: Supplementary file 8 — Supplementary material 8 (TIFF 50617 kb) [file 401_2016_1663_MOESM8_ESM.tif]

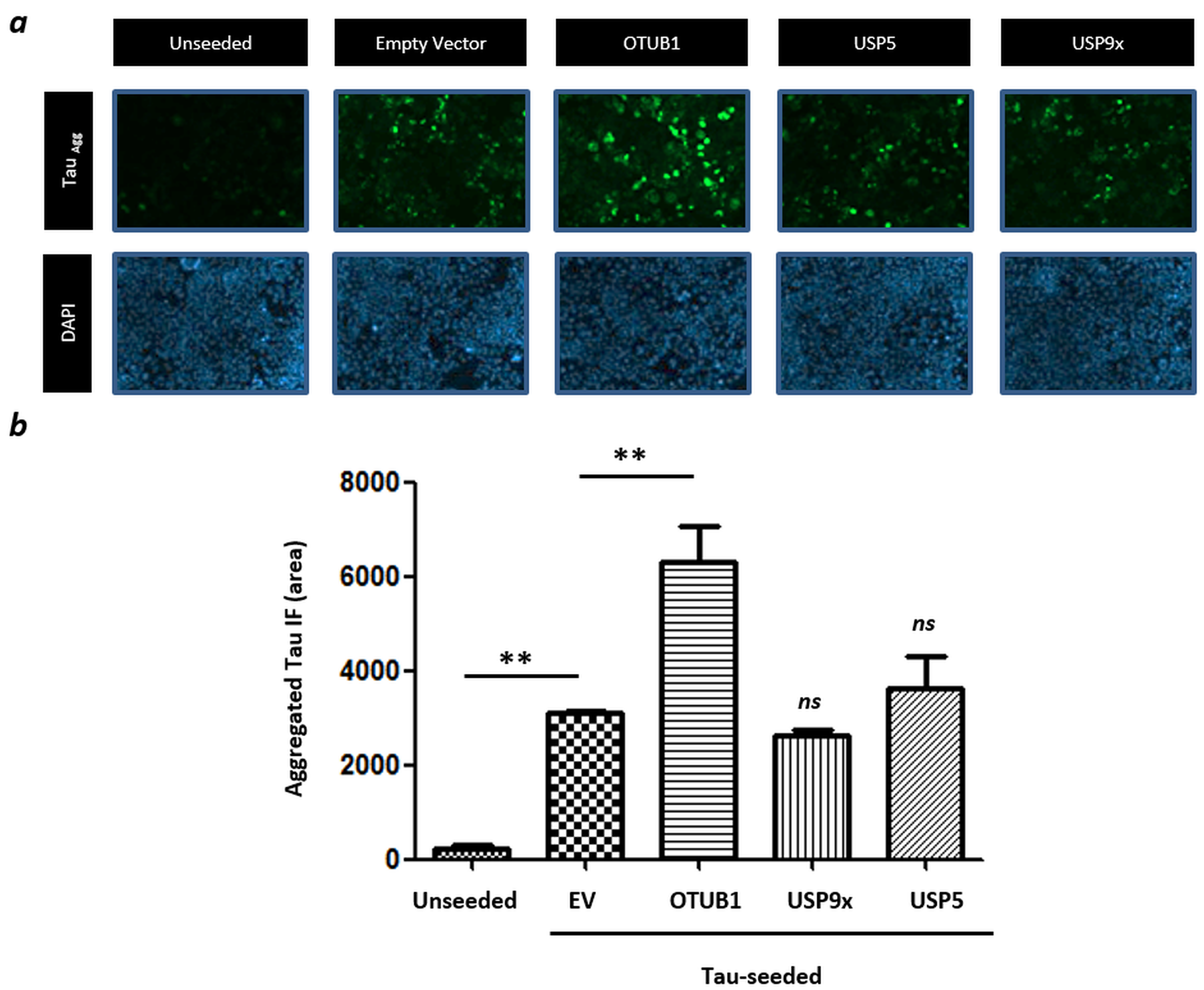

Supplement: Supplementary file 9 — Supplementary material 9 (TIFF 34212 kb) [file 401_2016_1663_MOESM9_ESM.tif]

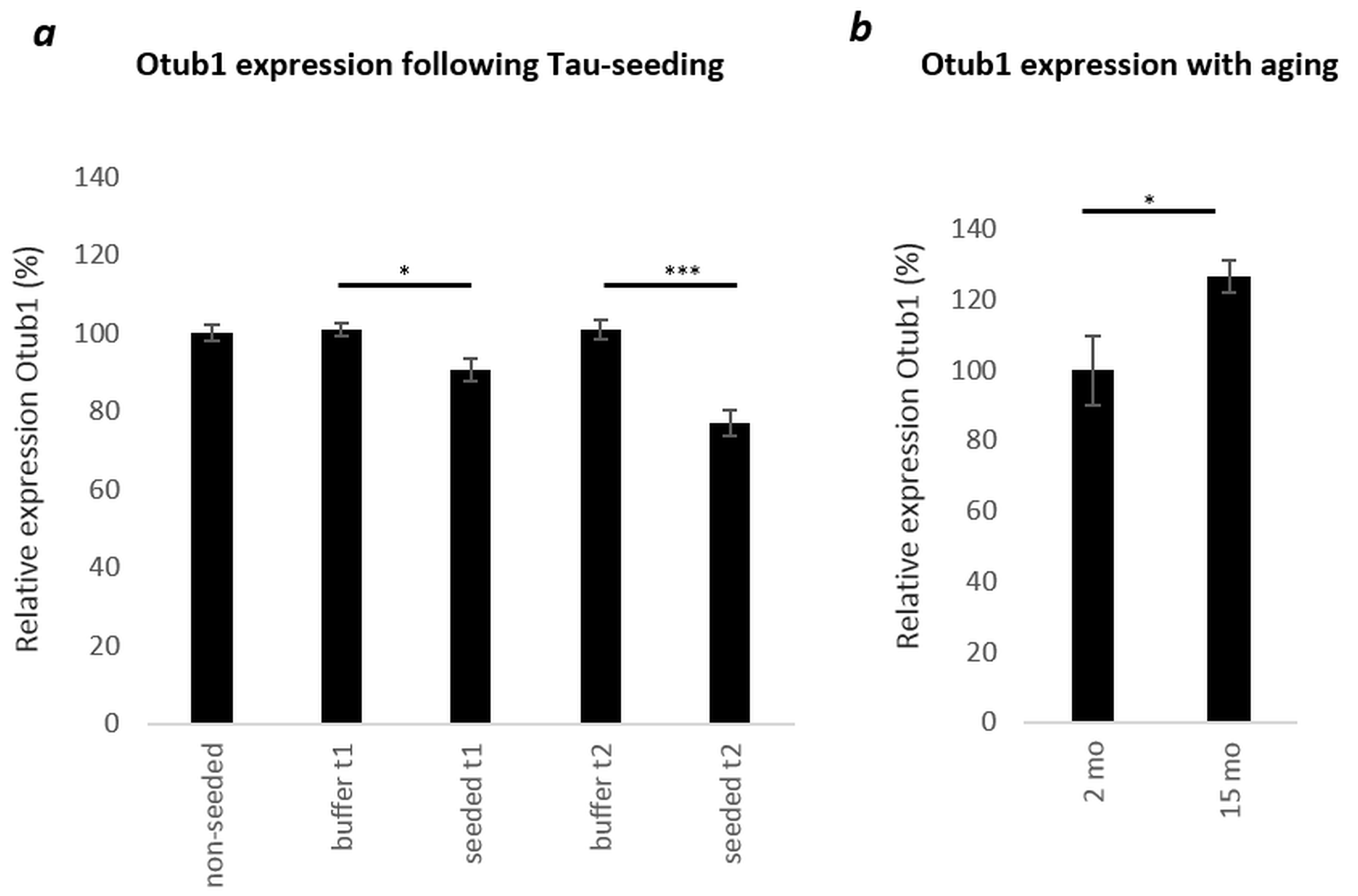

Supplement: Supplementary file 10 — Supplementary material 10 (TIFF 22053 kb) [file 401_2016_1663_MOESM10_ESM.tif]
